# *Plasmodium falciparum* origin recognition complex subunit 5: functional characterization and role in DNA replication foci formation

**DOI:** 10.1111/j.1365-2958.2008.06316.x

**Published:** 2008-06-19

**Authors:** Ashish Gupta, Parul Mehra, Suman Kumar Dhar

**Affiliations:** Special Centre for Molecular Medicine, Jawaharlal Nehru UniversityNew Delhi 110067, India

## Abstract

The mechanism of DNA replication initiation and progression is poorly understood in the parasites, including human malaria parasite *Plasmodium falciparum*. Using bioinformatics tools and yeast complementation assay, we identified a putative homologue of *Saccharomyces cerevisiae* origin recognition complex subunit 5 in *P. falciparum* (PfORC5). PfORC5 forms distinct nuclear foci colocalized with the replication foci marker proliferating cell nuclear antigen (PfPCNA) and co-immunoprecipitates with PCNA during early-to-mid trophozoite stage replicating parasites. Interestingly, these proteins separate from each other at the non-replicating late schizont stage, citing the evidence of the presence of both PCNA and ORC components in replication foci during eukaryotic DNA replication. PfORC1, another ORC subunit, colocalizes with PfPCNA and PfORC5 at the beginning of DNA replication, but gets degraded at the late schizont stage, ensuring the regulation of DNA replication in the parasites. Further, we have identified putative PCNA-interacting protein box in PfORC1 that may explain in part the colocalization of PfORC and PfPCNA. Additionally, use of specific DNA replication inhibitor hydroxyurea affects ORC5/PCNA foci formation and parasitic growth. These results strongly favour replication factory model in the parasites and confer great potential to understand the co-ordination between ORC and PCNA during eukaryotic DNA replication in general.

## Introduction

In eukaryotes, DNA replication takes place in subnuclear foci termed as replication foci (Gilbert, 2001). These foci contain several DNA replication accessory factors that include, but are not limited to, DNA polymerases, proliferating cell nuclear antigen (PCNA), DNA ligase, DNA methyltransferase, etc. (Leonhardt *et al*., 2000). It is believed that each focus point harbours several replication forks and the number may vary from 10 to 40 in metazoan cells (Berezney *et al*., 2000; Cook, 2001). Analysis of a stable mammalian cell line expressing low level of GFP-PCNA and a similar strategy using GFP-PCNA expression in *Saccharamyces pombe* reveal that the pattern and distribution of replication foci change with progression through S phase in eukaryotes (Leonhardt *et al*., 2000; Meister *et al*., 2007).

A six-protein origin recognition complex (ORC), conserved from yeast to mammals, binds to the DNA replication sites followed by concomitant binding of other replication initiation and elongation factors like cdc6, minichromosome maintenance proteins, cdt1, cdc45, PCNA, DNA polymerase, etc. (Bell and Dutta, 2002). Among these proteins, PCNA is considered to be the marker for active replication foci as it interacts and enhances the processivity of the DNA polymerase enzymes (Moldovan *et al*., 2007). Attempts for complete colocalization of ORC subunits with replication foci containing PCNA has not been successful yet, probably because of the fact that ORC subunits have different functions other than DNA replication. While ORC1 might have role in heterochromatin silencing, ORC2 and ORC6 are involved in centrosome copy number control and cytokinesis respectively (Pak *et al*., 1997; Prasanth *et al*., 2002; Prasanth *et al*., 2004).

Little is known regarding the control of DNA replication in human malaria parasite *Plasmodium falciparum*. During blood stage asexual development, each haploid merozoite following invasion in red blood cell forms ring stage parasite followed by trophozoite and multinucleated mature schizont before releasing new merozoites in a cycle of ∼48 h. Based on studies on the incorporation of radiolabelled precursors during *in vitro P. falciparum* culture, it has been suggested that the majority of DNA synthesis starts in synchronized *P. falciparum* culture ∼28–31 h after merozoite invasion and DNA content then continues to increase for around 8–10 h (Inselburg and Banyal, 1984; Graeser *et al*., 1996; Gantt *et al*., 1998; Arnot and Gull, 1998) that include several rounds of DNA replication. This is followed by the schizogony where four to five nuclear divisions take place in the common cytosol before the nuclei and other organelles are segregated into new merozoites (Leete and Rubin, 1996).

The biochemistry and enzymology of nuclear *Plasmodium* DNA replication has been restricted to the cloning and characterization of some replication factors like PfORC1, PfMCMs (mini-chromosome-maintenance proteins), PfPCNA, PfRPA and few DNA polymerase enzymes (Ridley *et al*., 1991; Kilbey *et al*., 1993; Patterson *et al*., 2002; Voss *et al*., 2002; Mehra *et al*., 2005; Gupta *et al*., 2006; Patterson *et al*., 2006; Nunthawarasilp *et al*., 2007). Few *Plasmodium* homologues of cyclins and cdk-like kinases have been reported (Doerig *et al*., 2002; Ward *et al*., 2004). Neither their roles in *Plasmodium* DNA replication nor their cellular targets have been established yet.

Because of the scarcity of knowledge regarding the DNA replication machinery in *Plasmodium*, detailed analysis and understanding of the replication components are important. This may also be fruitful to find suitable targets for much needed novel intervention strategies. We are particularly interested to identify new replication factors in *Plasmodium* and their role in DNA replication foci formation in *P. falciparum* during development. To follow replication foci formation and progression during parasite development, we have used two marker proteins, namely ORC component and PCNA respectively. We have recently reported the cloning and functional characterization of *P. falciparum* ORC1 homologue that is essential for initiation of DNA replication (Mehra *et al*., 2005). PfORC1 is expressed in the nucleus during trophozoite and early schizont stages and the recombinant protein shows ATPase activity, the hallmark of ORC function. Apart from ORC1, ORC5 has also been reported to contain ATP binding activity, essential for ORC activity both *in vivo* and *in vitro* (Takahashi *et al*., 2004; Giordano-Coltart *et al*., 2005). In addition to its conserved role in DNA replication initiation, ORC1 has also been implicated in heterochromatin silencing in higher eukaryotes (Pak *et al*., 1997). After careful analysis of *P. falciparum* genomic database, we have identified a putative PfORC5 homologue that gave us the opportunity to track the ORC binding sites in *P. falciparum.*

In order to track the formation and progression of replication foci during different erythrocytic developmental stages in *P. falciparum*, we used PfPCNA1 as a marker for replication foci. The cloning and characterization of PfPCNA1 was initially described in 1993 (Kilbey *et al*., 1993). Although only one form of PCNA is found in *S. cerevisiae* and mammals, two or three types of PCNA have been reported recently in apicomplexan *Toxoplasma gondii*, archaeans, higher plants and *Drosophila* (Hata *et al*., 1992; Guerini *et al*. 2000; Daimon *et al*., 2002; Ruike *et al*., 2006). These different forms of PCNA may form homo- or hetero-trimeric sliding clamps with their involvement in DNA replication or repair or both. Interestingly, two PCNA homologues have been reported (PfPCNA1 and PfPCNA2 respectively) in *Plasmodium* (Li *et al*., 2002; Patterson *et al*., 2002). Although both the PCNA are expressed during asexual and sexual stages, only PfPCNA1 seems to contain all the conserved motifs for PCNA, including the conserved helix-turn-helix DNA-binding domain at the N terminus. Moreover, in a phylogenetic analysis, PfPCNA1 is grouped with *T. gondii* PCNA1 (Li *et al*., 2002) that has been suggested to be the major replisomal PCNA using biochemical analysis and genetic tools (Guerini *et al*., 2005). Biochemical experiments suggest that PfPCNA1 and PfPCNA2 are part of the same replisome complex (Patterson *et al*., 2002). Therefore, we have considered PfPCNA1 as the marker for replication foci in *P. falciparum* and hereafter it will be termed as PfPCNA.

Using specific antibodies against PfORC1, PfORC5 and PfPCNA, here we show that PfORC components and PfPCNA co-immunoprecipitate with each other and they form distinct colocalized foci following immunofluorescence experiments just at the onset of DNA replication in the early trophozoite stage parasites. As the DNA synthesis progresses, two key changes take place in the replication factories. PfORC5 and PfPCNA foci slowly dissociate from each other while PfORC1 is degraded in a proteasome-mediated pathway- suggesting a regulatory role of PfORC1 during blood stage parasite development. We suggest that a putative PCNA-interacting protein motif (PIP) identified in PfORC1 and other ORC1 homologues may facilitate the complex formation among ORC components and PCNA during DNA replication. Use of DNA replication inhibitor hydroxyurea affects parasite growth and replication foci formation. These results illustrate the conservation of factory model of replication in *Plasmodium* and confer a great potential to understand the importance of ORC proteins for replication foci formation and progression during S phase in general.

## Results

### Cloning, amino acid sequence analysis and expression of putative homologue of PfORC5

Upon blast search using full-length as well as C- and N-terminal regions of yeast and human ORC5 proteins as queries, one open reading frame (ORF) (PFB0720c) showed ∼20% identity and ∼43% homology with the yeast counterpart at the carboxyl-terminal region. Interestingly, HsORC5p shows ∼24% identity (∼48% similarity) with ScORC5p (Quintana *et al*., 1998) that is comparable with the homology of PFB0720c C-terminal region with ScORC5. This ORF has been annotated as conserved hypothetical protein in PLASMODB although the corresponding homologues in *Plasmodium vivax* (Pv002750) and *Plasmodium yoelii* (PY01116) have been annotated as putative ORC5 homologues. The average length of ORC5 in other eukaryotes is between ∼430 and 480 residues whereas that of PFB0720c is 899 residues with an unusual long N-terminal extension containing two asparagine/aspartic acid/lysine repeat-rich regions (Fig. S1). This is not unusual feature for *Plasmodium* proteins as reported earlier (Singh *et al*., 2004), including the largest subunit of ORC, PfORC1 that also shows a long N-terminal extension with asparagines and lysine repeat residues (Mehra *et al*., 2005). Excluding the length of the N-terminal extension region with the repeat sequences, the length of C-terminal region of PFB0720c is ∼440 amino acid residues, within the range of ORC5 proteins in other species.

Full-length putative PfORC5 gene was amplified by polymerase chain reaction using two specific primers (P1 and P2, Table S1, supplementary information) and 3D7 genomic DNA. Following PCR, a single product (∼2.7 kb) was obtained (as shown in Fig. S2A) that was cloned into pET_28a_ vector and it was subsequently sequenced using suitable overlapping primers.

The alignment of amino acid sequence of putative PfORC5 homologue with *S. cerevisiae* ORC5 showed many interesting features (Fig. S1). A putative nucleotide binding domain (306–310 residues), one of the hallmarks of the ORC5 proteins, was found between two asparagine and aspartic acid-rich regions at the N terminus (repeat regions I and II respectively, Fig. S1). The ORC5 homology domain was found at the carboxyl-terminal region (484–899 residues). A putative nuclear localization signal-containing motif was also identified at the N-terminal region of PfORC5.

**Fig. 1:**
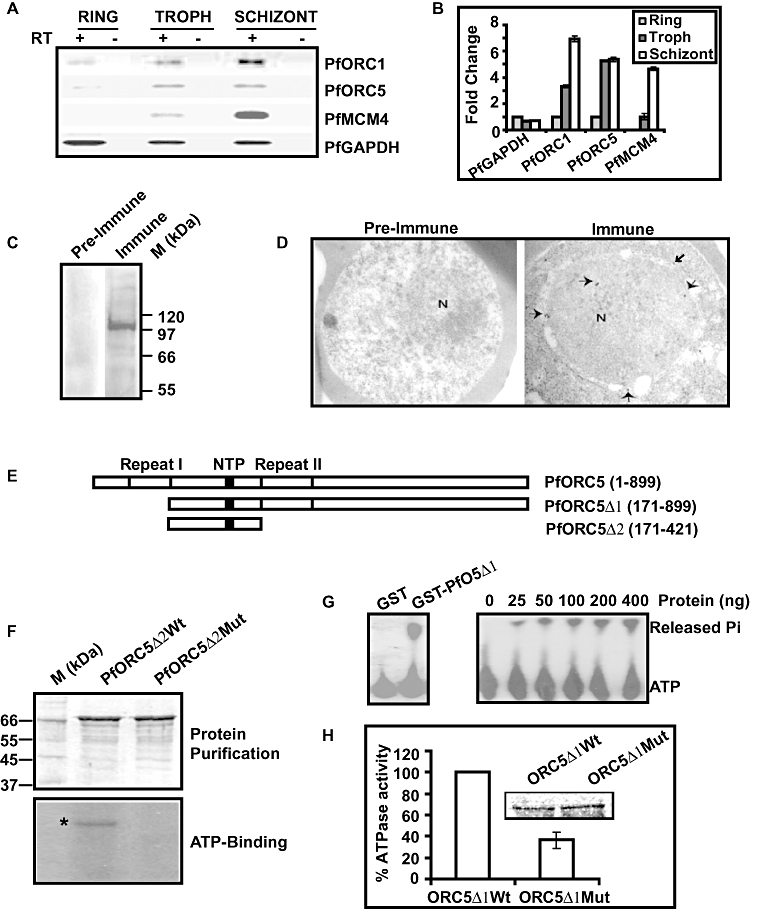
Expression of PfORC5 at the transcript and protein level and ATP binding/hydrolysis activity of PfORC5. A. RT-PCR analysis of PfORC1, PfORC5, PfMCM4 and control GAPDH using cDNA obtained from different developmental stages. RT (+) and RT (−) lanes indicate the PCR products obtained from cDNA prepared either in the presence or absence of reverse transcriptase enzyme respectively. B. Fold changes in expression of different genes at different stages. The relative intensity of each band was calculated using densitometry scanning and the absolute values were calculated by normalizing against background intensity. Each experiment was repeated three times and the values obtained including the standard deviations were plotted graphically. C. Western blot analysis to analyse the expression of PfORC5 at the protein level using either anti-PfORC5 or pre-immune sera. Molecular mass markers are shown on the right. D. Immunoelectron-microscopic localization of PfORC5 within paraffin-embedded parasite sections using either pre-immune or anti-PfORC5 antibodies respectively. Arrows indicate gold particle depositions and ‘N’ stands for nucleus. E. Schematic diagram of wild type or different deletion mutants of PfORC5 or full-length *S. cerevisiae* ORC5. Amino acid co-ordinates were shown on the right. F. ATP binding activity of PfORC5. One microgram of the MBP-fused PfORC5Δ2Wt or PfORC5Δ2mut proteins were incubated with [α-^32^P]-dATP and was further cross-linked using UV light as described in *Experimental procedures*. Upper panel shows the SDS-PAGE analysis of these proteins. The molecular mass markers are shown on the left. The bottom panel shows the autoradiogram of the above gel. G. ATPase activity of GST-PfORC5Δ1. ATPase assays were performed either using GST-PfORC5Δ1 or GST as a control. The positions of the radiolaelled ATP and the released Pi are marked. The right panel shows the ATPase activity of GST-PfORC5Δ1 with increasing amount of protein. H. Comparison of the ATPase activity of wild type and mutant proteins. ATPase assays were performed using 100 ng of GST-PfORC5Δ1Wt or GST-PfORC5Δ1Mut and phosphate release was quantified and plotted graphically with standard deviation after repeating the experiments for three times. The inset shows the purification of the wild type and mutant forms of the proteins.

In order to investigate the expression pattern of PfORC5 during asexual erythrocytic stages, semi-quantitative RT-PCR analysis was performed using cDNA isolated from synchronized ring, trophozoite and schizont stage parasites and specific primers (P15 and P16, Table S1, supplementary information) as shown in the schematic diagram of PfORC5 (Fig. S2B). Total RNA was treated with DNase I before processing for cDNA preparation either in the presence or absence of reverse transcriptase enzyme (+RT and -RT lanes respectively, Fig. 1A). Comparison of RT products from different erythrocytic developmental stages reveals that the expression level of PfORC5 goes up several folds during trophozoite and schizont stages in comparison with ring stage whereas the control PfGAPDH expression pattern does not fluctuate to the similar extent during all three stages (Fig. 1B). PfORC1 and PfMCM4, the other two members of pre-replication complex (pre-RC) are also expressed mostly in the trophozoite and schizont stage parasites similar to PfORC5 expression pattern (all the primers for RT-PCR analysis are listed in Table S1, P15–P22). RT-PCR analysis of PfORC5, PfORC1 and PfMCM4 supports the published microarray data, confirming a peak of transcription coincident with onset of DNA replication (Bozdech *et al*., 2003).

To investigate the expression level of PfORC5 at the protein level, two polyclonal antibodies were raised in the rabbit and mouse, respectively, against C-terminal region of PfORC5 (PfORC5C1 and PfORC5C2 respectively) as shown in Fig. S2B. Polyclonal antibodies raised in rabbit predominantly recognized a band around molecular mass of ∼105 kDa following Western blot analysis of parasite lysate (Fig. 1C). Pre-immune sera under the same experimental conditions do not recognize any such band, suggesting that these antibodies are specific against PfORC5. Polyclonal antibodies raised in mouse also recognized a similar band in Western blot experiments (Fig. S2C).

Further, to investigate the subcellular localization of PfORC5 expression within the parasite, electron microscopic analysis of sections of trophozoite stage parasites were performed using either rabbit pre-immune sera or immune sera raised against PfORC5. Analysis of gold particles deposition within the parasite clearly shows that PfORC5 is expressed primarily within the nucleus and not in the cytoplasm or red blood cell (RBC) (Fig. 1D). Several nuclei were scanned and it was found that the gold particles were distributed all over the nuclei. On average, approximately six to eight particles were found in the trophozoite stage nuclei in each section. Pre-immune sera under the same experimental conditions do not show any such gold particle deposition, suggesting the specificity of these antibodies to detect PfORC5 in the nucleus.

### ATP binding, ATPase activity of PfORC5 and functional complementation in *S. cerevisiae*

One of the hallmarks of ORC5 is its affinity to ATP because of the presence of Walker A NTP binding domain (Bell, 2002). Mutation in the Walker A domain of *S. cerevisiae* ORC5 causes temperature-sensitive growth phenotype, suggesting that ATP binding to ORC5 is important for chromosomal DNA replication (Takahashi *et al*., 2004). *In vitro*, recombinant ORC containing a mutation in the ATP binding domain of HsORC5 shows reduced DNA binding affinity (Giordano-Coltart *et al*., 2005). PfORC5 contains a putative Walker A domain at the N terminus of the protein (^306^GMGKT^310^) flanked by repeat regions I and II (Fig. S1E).

To investigate the ATP binding and ATPase activity of PfORC5, we have purified different domains of PfORC5 (Fig. 1E). Unfortunately, we failed to express full-length PfORC5 because of the presence of asparagines and aspartic acid repeat regions at the extreme N-terminal region. However, we have been able to express PfORC5Δ1 (excluding the N-terminal repeat regions) and PfORC5Δ2 (containing the NTP-binding domain) (Fig. 1E). First, we investigated the ATP binding property of PfORC5 using recombinant PfORC5Δ2 because of its better yield over PfORC5Δ1. We also made a mutant form of the protein by introducing a point mutation at the Walker A domain (K to A). Equal amount of wild type and mutated form of the proteins were incubated with [α-^32^P] dATP and subjected to UV cross-linking analysis. Following autoradiography, wild-type PfORC5Δ2 was found to be in ATP-bound form whereas the mutated protein did not bind to ATP under the same experimental conditions (Fig. 1F).

Although ORC1 has been reported to contain ATPase activity, no such activity has been attributed to ORC5 yet (Mehra *et al*., 2005). This is due to the absence of a clear WALKER B domain designated as nucleotide hydrolysis domain in ORC5 homologues from yeast to mammals. However, there is always a possibility of a cryptic WALKER B domain present in these proteins. In order to find out whether PfORC5 contains ATPase activity, release of inorganic phosphate (Pi) from [γ-^32^P] ATP was monitored in the presence of GST-PfORC5Δ1 or GST as a control. The recombinant protein showed ATPase activity in a concentration-dependent manner whereas the control GST protein did not show any such activity under the same experimental conditions (Fig. 1G). A mutant form of PfORC5Δ1, containing a point mutation in the ATP binding domain (K to A within the ‘GMGKT’ NTP binding motif) showed drastically reduced ATPase activity when compared with that of wild-type protein (Fig. 1H). The residual activity in PfORC5Δ1Mut could be due to the presence of some minor ATPase contaminant in the protein preparation. Identification of WALKER B NTP hydrolysis domain in PfORC5 will be helpful to ascertain the ATPase activity of PfORC5.

**Fig. 2:**
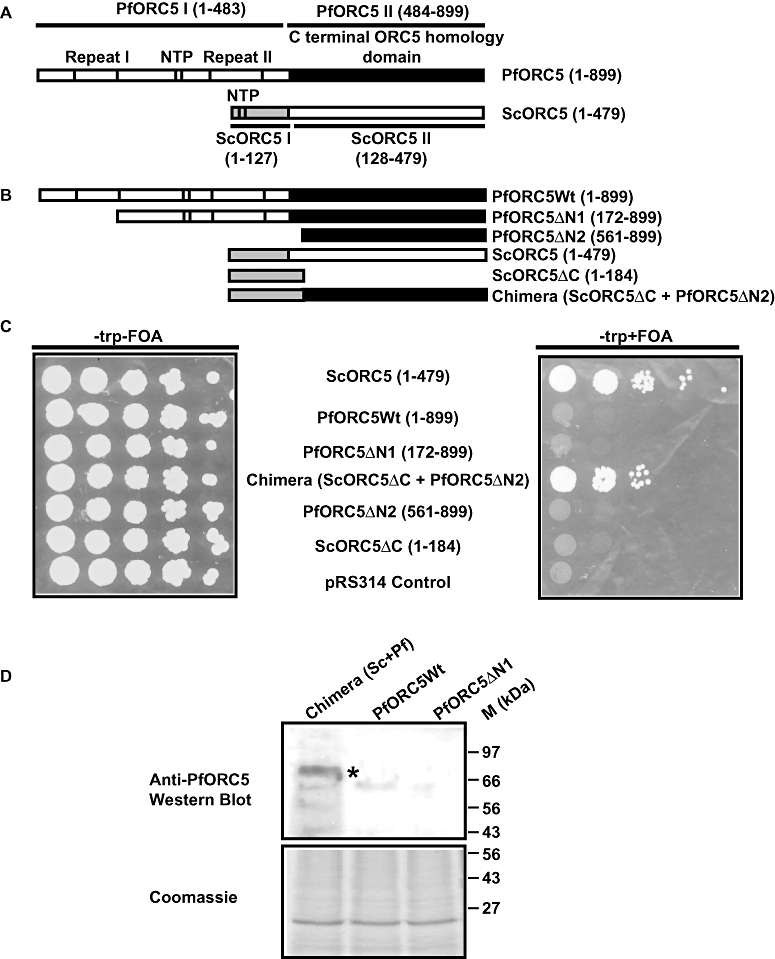
Complementation of PfORC5 in *S. cerevisiae*. A. Schematic diagrams of PfORC5 and ScORC5. The repeat regions (I and II) and NTP binding domains are shown as open boxes whereas the filled box shows the C-terminal ScORC5 homology region. The grey box in ScORC5 shows the N-terminal region containing NTP binding domain. Both PfORC5 and ScORC5 can be divided into two parts (I and II) respectively. B. The schematic diagrams show different regions of PfORC5 and ScORC5 or the chimera containing N terminus of ScORC5 and C terminus of PfORC5 as used for complementation assay. The rationale for chimera construction has been described in *Results* section and supplementary method section. C. A swapper strain of *S. cerevisiae ORC5* with the deletion of chromosomal copy of *ScORC5* gene and maintaining the same gene with a plasmid containing *ura* marker was transformed with either wild-type or different chimeras of *PfORC5* and *ScORC5* (with *trp* selection) either in the presence or absence of 5-fluoroorotic acid. Growth of these transformants was followed by spot test using serial dilutions. D. Western blot analysis to show the expression of different constructs as used in the complementation assay. Anti-PfORC5 polyclonal antibodies were used for Western blot analysis. The coomassie-stained gel following protein transfer on PVDF membrane is shown as loading control. * indicates the expression of chimera protein in yeast. The molecular mass marker is shown on the right.

To further prove that the putative PfORC5 is a true ORC5 homologue, we performed functional complementation in yeast using either full-length or deletion mutant of PfORC5 or chimera constructs of ScORC5 and PfORC5 fusing N- and C-terminal of these respective proteins. We adopted the chimera approach because of the presence of two repeat regions (I and II respectively, Figs S1 and 2A) at the N terminus of PfORC5 that may affect the expression of full-length PfORC5 in yeast. We decided to fuse the N-terminal region of ScORC5 containing NTP binding domain with the C-terminal homology region of PfORC5 (Fig. 2A). While doing the same, we also considered the secondary structure of both PfORC5 and ScORC5 so that we do not delete helix or beta sheet regions of these proteins. We used the overlapping PCR method to construct the chimeras (Horton *et al*., 1989). This method allowed us to fuse ScORC5ΔC (1–184) and PfORC5ΔN2 (561–899) to get Chimera ORC5 (ScORC5ΔC + PfORC5ΔN2). The strategy for making the chimera is shown in schematic diagrams (Fig. 2A and B) and the detailed methods are described in supplementary data (*Experimental procedures* section, complementation of yeast *ORC5* mutant strain). An *ORC5* mutant haploid strain of *S. cerevisiae* (ySPB5.11, with *S. cerevisiae* W303 background, a kind gift from Dr Steve Bell, MIT, USA) with a deletion of chromosomal copy of *ORC5* and having wild-type *ORC5* gene in a plasmid containing *ura3* marker was used for complementation studies. This yeast strain was transformed with plasmid constructs containing either full-length PfORC5 or ScORC5 or PfORC5ΔN1 (172–899 aa) or the chimera construct, or N-terminal and C-terminal domains of ScORC5 and PfORC5 alone respectively, or pRS314 empty vector alone under galactose-inducible promoter with a tryptophan marker. Following transformation, the transformants were grown either in the absence or presence of 5-fluoroorotic acid for the selection of viable yeast cells in minimal media without tryptophan. We find that full-length ScORC5 complements this mutant yeast strain in the presence of 5-FOA whereas full-length PfORC5 and PfORC5ΔN1 cannot rescue these cells under the same experimental conditions (Fig. 2C). Interestingly, the chimera containing N terminus of ScORC5 and C terminus of PfORC5 complements these cells whereas only N-terminal region of ScORC5 or only C-terminal region of PfORC5 or the empty vector pRS314 cannot rescue these cells (Fig. 2C). The inability of PfORC5 to complement the yeast mutant strain is due to the presence of repeat regions at the N terminus affecting the expression of full-length protein or PfORC5ΔN1 in yeast. It was further confirmed by Western blot analysis using the lysate obtained from yeast cells containing either wild-type PfORC5 or PfORC5ΔN1 or chimera constructs. Anti-PfORC5 antibodies specifically recognized a band in chimera lane only with a molecular mass of ∼70 kDa (consistent with the molecular mass of chimera construct) (upper panel, Fig. 2D). The coomassie-stained gel confirms the presence of equal amount of protein in all the lanes (lower panel, Fig. 2D).

The presence of C-terminal ORC5 homology region in PFB0720c, the conserved and functional ATP binding domain at the N terminus of PFB0720c and the ability of the chimera construct to functionally complement *ScORC5* mutant yeast strain clearly suggest that the putative PfORC5 is a true homologue of ORC5.

### PfORC5 forms distinct foci within nuclei during intraerythrocytic developmental stages

In an effort to investigate subcellular localization and the pattern of PfORC5 expression during intraerythrocytic developmental stages, we performed thorough immunofluorescence analysis at different time points in a synchronized parasite population using immuno-affinity-purified anti-PfORC5 antibodies. We also used anti-PfPCNA antibodies to locate the active replication foci in these cells as PCNA has been widely used as a marker for growing replication forks (Leonhardt *et al*., 2000; Meister *et al*., 2007).

**Fig. 3:**
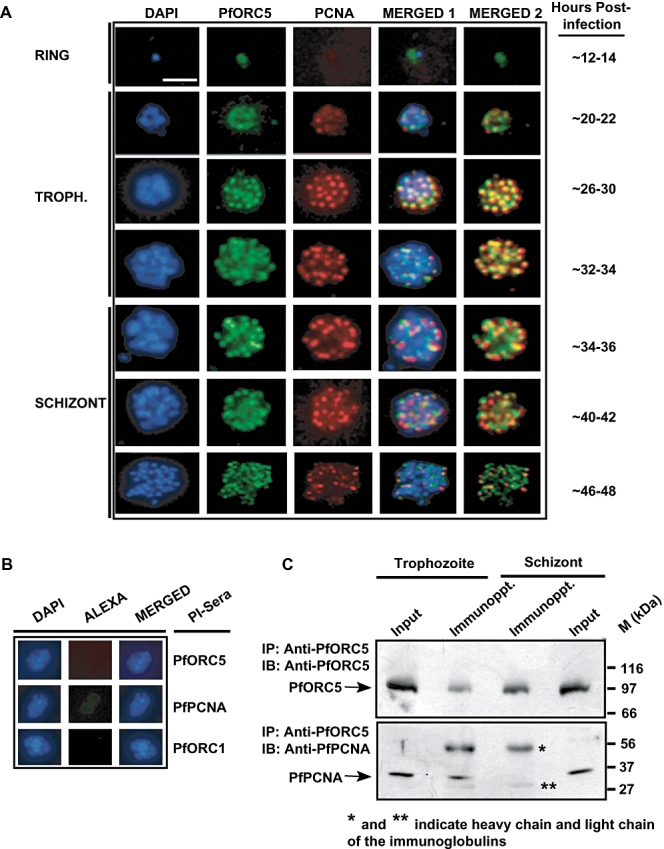
Colocalization and interaction between PfORC5 and PfPCNA. A. Immunofluorescence assay to show colocalization of PfORC5 and PfPCNA as replication foci marker. Glass slides containing thin smears of *P. falciparum*-infected erythrocytes from the different erythrocytic stages were incubated with affinity-purified rabbit anti-PfORC5 and mouse anti-PfPCNA antibodies followed by respective secondary antibodies. Merged 1 panel shows the merged images of nuclear DAPI, green PfORC5 staining and red PfPCNA staining whereas merged 2 panel shows the merged images of green PfORC5 and red PfPCNA signals only. The bar as shown in the inset is equivalent to 3 μm. B. Immunofluorescence assay under the same experimental conditions as shown in A using pre-immune sera against respective antibodies. C. Co-immunoprecipitation of PfORC5 and PfPCNA from parasite extract obtained from synchronized parasite pellet collected from either trophozoite or late schizont stage parasites. The extracts were first immunoprecipitated (IP) with anti-PfORC5 antibodies followed by immunoblotting (IB) with either anti-PfORC5 (top panel) or anti-PfPCNA (bottom panel) antibodies. The input lanes show the presence of these proteins in both the stages. Arrowhead indicates the position of the respective proteins. Molecular mass markers are shown on the right.

Analysis of immunofluorescence images at different time points revealed the presence of PfORC5 at the ring and early trophozoite phase parasites with a diffused staining pattern. Interestingly, PfORC5 forms distinct foci within nuclei as the parasites mature further (mid-trophozoite phase parasites) (Fig. 3A). The size and the number of such foci increase as the parasites undergo further maturation until late schizont stage where the average size of such foci decreases compared with those found in mid-trophozoite and early schizont stages. When the same parasites were screened for PCNA (replication foci marker) signals, it showed somewhat diffused pattern during early trophozoite stage like PfORC5. As the parasites developed further into mid-trophozoite stages, PfPCNA showed distinct foci as PfORC5 and these dots mostly merged with PfORC5 dots as seen from the merged panels (Fig. 3A, panel 3). The number and average size of the PCNA foci also increased till mid-schizont stages and both these parameters decreased at the late schizont stages. Although the PfORC5 and PfPCNA foci merged mostly with each other at these stages, these foci slowly dissociated from each other as the parasites developed further and the majority of these spots separated from each other at the late schizont stage (Fig. 3A, panel 7). Please see enlarged and high-resolution Fig. S3A for the expression pattern of these proteins at late schizont stage. In a control set of experiments, none of the pre-immune sera show any immunofluorescence signal under the same experimental conditions, confirming the specificity of these antibodies (Fig. 3B).

It will be interesting to see whether the replication foci formation and progression are correlated with the extent of DNA replication through intra-erythrocytic development cycle. Additionally, the immunofluorescence studies presented here are based on the slides containing parasite smears that have been fixed using acetone-methanol fixation method (please see the *Experimental procedures* section for details). The slides were washed very extensively during immunofluorescence assay (IFA) to avoid any background staining. Although this method gives us distinct pattern of replication foci formation, the DAPI (9,6-diaminido-2-phenylindole) shows diffused staining pattern. In order to avoid any bias that may arise because of a particular method of fixation, we repeated the IFA using synchronized parasites that were fixed using paraformaldehyde (PFA). For this purpose, we synchronized the 3D7 parasites at the ring stage and the same was divided into four aliquots and they were allowed to grow further. The growth of the parasites was monitored time to time. The parasites were harvested at ring, trophozoite, early schizont and late schizont stages. The glass slides were made to confirm their stages by giemsa staining (Fig. S4A) and they were fixed with PFA and preserved for further IFA analysis. Total genomic DNA was isolated in each case and they were analysed by agarose gel electrophoresis (Fig. S4B). The total DNA was further quantified spectrophotometrically and plotted accordingly (Fig. S4C). We find that as the parasites grow, the total DNA content increases many folds from ring to schizont stages, reflecting a huge amount of concomitant DNA replication with growth progression.

Further analysis of the parasites obtained from the same stages as described above following IFA experiments using anti-PfORC5 and anti-PfPCNA antibodies suggests a similar pattern of replication foci formation and progression during development. Both PfPCNA and PfORC5 formed colocalized distinct foci during early-to-mid replicating trophozoite stages. These foci started dissociating from each other with further growth progression and finally they separated completely during late schizont stage as shown earlier (Fig. S5). The average number of PfPCNA or PfORC5 foci or the colocalized foci and their pattern were found to be similar as compared with those shown in Fig. 3 by methanol-acetone fixation method.

Although ORC components colocalize with PCNA during S phase to a limited extent in mammalian cells (Prasanth *et al*., 2004), no biochemical evidence is available yet to prove that these proteins are truly the components of replication foci.

To verify whether ORC components and PCNA are truly the members of replication factories, we performed immunoprecipitation reactions using parasite extracts and antibodies against PfORC5 and PfPCNA. Both PfPCNA and PfORC5 can be immunoprecipitated from mixed trophozoite stage parasite extract using immune sera but not with pre-immune sera (Fig. S3B). To find out whether these proteins co-immunoprecipitate with each other, we first immnuprecipitated PfORC5 from the parasite extract derived from either mixed stage trophozoites or late schizont stage parasites followed by immunoblotting using anti-PfORC5 or anti-PfPCNA antibodies respectively (Fig. 3C, top and bottom panels). We found that PfORC5 was immunoprecipitated from both trophozoite and schizont stages (Fig. 3C, top panel). Interestingly, we observed that PfPCNA was co-immunoprecipitated with PfORC5 during trophozoite stage only but not at the late schizont stage. PfPCNA was expressed at both the stages to the similar extent as shown by the input lanes (Fig. 3C, bottom panel). These results confirm the colocalization of PfORC5 and PfPCNA during trophozoite stages but not at the schizont stages.

### PfORC1, another member of the ORC colocalizes with PfORC5 and PfPCNA but gets degraded at the late schizont stage

**Fig. 4:**
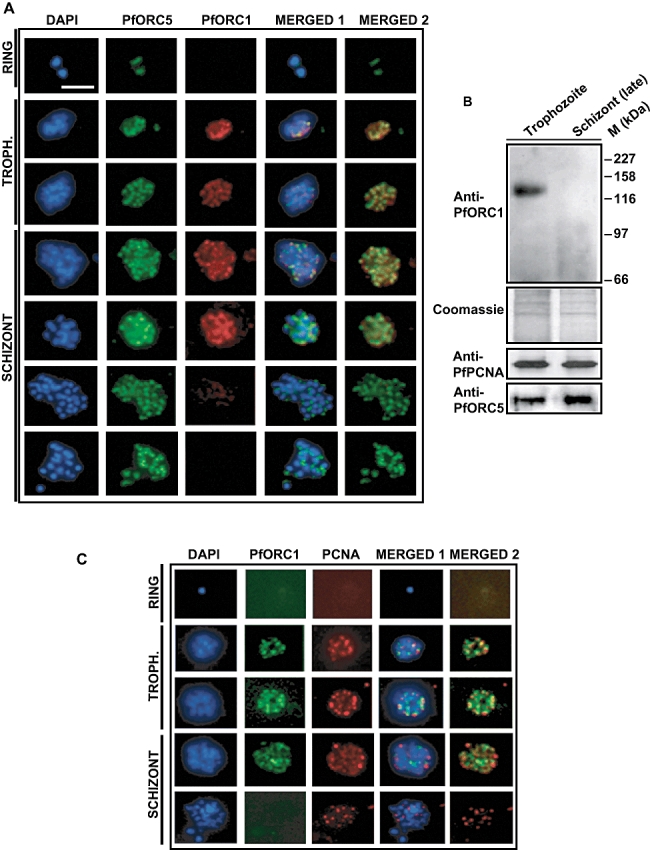
Colocalization pattern of PfORC5 and PfORC1 during different erythrocytic stages. A. Immunofluorescence assay to show expression pattern of PfORC5 and PfORC1 during different development stages. Affinity-purified rabbit anti-PfORC5 and mouse anti-PfORC1 antibodies were used as primary antibodies followed by respective secondary antibodies. Merged 1 panel shows the merged images of nuclear DAPI, green PfORC5 and red PfORC1 signals whereas merged 2 panel shows the merged images of green PfORC5 and red PfORC1 signals. The bar is equivalent to 3 μm. B. Western blot analysis to compare the expression of various replication initiation proteins during trophozoite and late schizont stages. Equivalent amount of parasite pellet obtained from the above two stages were boiled in SDS-PAGE loading buffer followed by Western blot analysis using either anti-PfORC1 or anti-PfPCNA or anti-PfORC5 antibodies. Molecular mass markers are shown on the right. The membrane following Western blot was coomassie-stained to show the loading control. C. Colocalization pattern of PfORC1 and PfPCNA during parasite development. Immunofluorescence assay was performed as described earlier using rabbit anti-PfORC1 and mouse anti-PfPCNA antibodies. The bar as shown in the inset is equivalent to 3 μm.

Although ORC functions collectively as a replication initiator protein, different components of ORC are regulated differentially in a cell cycle manner. The level of ORC2–5 does not change through cell cycle whereas ORC1 comes on and off the chromatin in a cell cycle-regulated manner in mammalian cells (Méndez *et al*., 2002). In hamster cells, ORC1 is dissociated from chromatin as cells enter S phase, transformed into a mono- or diubiquitinated form, followed by deubiquitination and re-binding to chromatin during the M-to-G1 transition (Li and DePamphilis, 2002). To investigate the fate of PfORC1 during different developmental stages, we performed immunofluorescence experiments as shown in Fig. 4A using rabbit anti-PfORC5 and mouse anti-PfORC1 antibodies simultaneously. PfORC5 showed same pattern of foci formation and progression as shown earlier (Fig. 3A). PfORC1 was not detected in ring stage parasites followed by a diffused pattern in early trophozoite parasites. During mid-trophozoite stages, PfORC1 also showed multiple foci that mostly merged with PfORC5 foci (Fig. 4A, panel 3). These merged patterns continued till mid-schizont stages until PfORC1 signal was completely diminished in the late stage parasites (Fig. 4A, last two panels). These results indicate that PfORC1 is degraded at the late schizont stage parasites whereas PfORC5 still persists in a different compartment than PCNA.

To revalidate immunofluorescence data, we performed Western blot analysis using parasite extract derived from synchronized parasites either from mixed stage trophozoites or late stage schizonts. Western blot analysis detected the presence of PfORC1 in trophozoite stage but not in the late schizont stage whereas both PfORC5 and PfPCNA were present at both the stages (Fig. 4B). Coomassie-stained membrane following Western blot shows that the loading of proteins was comparable in both the lanes (Fig. 4B, second panel). These results strongly suggest that different ORC components are regulated differentially during development.

We further investigated colocalization pattern of PfORC1–PfPCNA during development. Co-immunofluorescence analysis using anti-PfORC1 and anti-PfPCNA antibodies showed that the majority of the ORC1 and PCNA foci merged with each other during trophozoite stages (Fig. 4C, panels 2 and 3). ORC1 signal was completely abolished during late schizont stages whereas PCNA foci could still be found during this stage (Fig. 4C, last panel).

### Identification of putative PIP motif in PfORC1

**Fig. 5:**
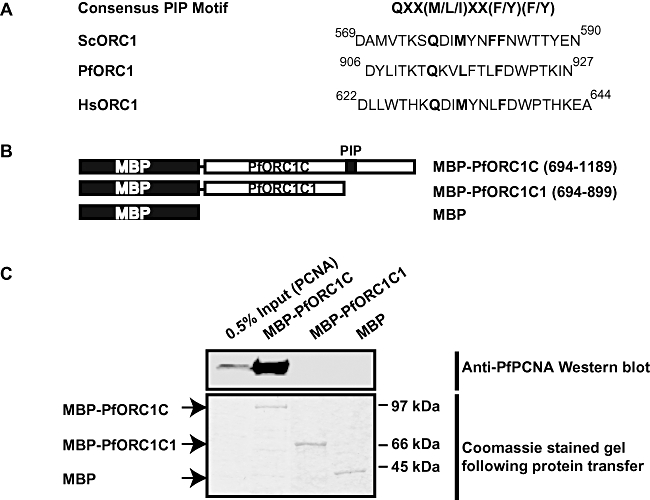
Identification of conserved PIP motif in ORC1. A. The consensus PIP motif is shown on the top. The putative PIP domains present in ScORC1, PfORC1 and HsORC1 are also shown. The conserved residues are marked in bold. B. The schematic diagrams of C-terminal region of PfORC1 with (PfORC1C) or without (PfORC1C1) PIP domain fused to MBP. The residues of PfORC1 taken in each construct are also shown. The PIP motif is marked as black box. C. Pull-down experiments using MBP-fused ORC1 constructs. Two microgram of His_6_-PCNA was incubated with equal amount of MBP beads containing MBP-ORC1C (+PIP motif) or MBP-ORC1C1 (−PIP motif) or MBP alone followed by stringent washing of the beads using buffer as mentioned in the *Experimental procedures*. The bound proteins were released by boiling in SDS-PAGE loading buffer followed by Western blot analysis using anti-PfPCNA antibodies. The input lane shows 0.5% PCNA. The bottom panel shows the loading control for pull-down experiments with MBP alone or other fusion proteins.

We have found most strikingly the complete colocalization of replication foci marker PCNA and ORC components at the onset of DNA replication. So far, attempts to colocalize these proteins completely in S phase have failed in yeast and mammalian cells. We have further shown that these proteins are part of the replication factory by co-immunoprecipitation experiments. PCNA interacts with its several partners (e.g. Fen1, p21, CDT1, MCM10, DNA ligase, etc.) through a conserved PIP motif present in these proteins (Arias and Walter, 2006; Das-Bradoo *et al*., 2006; Moldovan *et al*., 2007). We were curious to know whether PfORC components would contain such PIP box that would justify the colocalization of these proteins in replication foci. Surprisingly, we have identified a close match to the conserved PIP motif, QXX(M/L/I)XX(F/Y)(F/Y) in PfORC1 (Fig. 5A). Interestingly, we have also identified PIP motif both in human and yeast ORC1, suggesting that this domain might play a fundamental role in eukaryotic DNA replication. We further performed direct pull-down experiments using maltose-binding protein (MBP) beads fused to PfORC1C (C terminus of PfORC1 with PIP motif) or PfORC1C1 (C terminus of PfORC1 without PIP motif) or MBP alone along with soluble recombinant purified PfPCNA. Following pull-down experiments and Western blot analysis with anti-PfPCNA antibodies, we found that PCNA interacted specifically with PfORC1C with PIP motif but not with PfORC1C1 without PIP motif or MBP alone under the same experimental conditions (Fig. 5B and C). Together with *in vivo* colocalization and co-immunoprecipitation data, these *in vitro* pull-down experiments suggest that ORC may facilitate the recruitment of PCNA to the replication fork and it may reflect a general mechanism for eukaryotes as PIP motifs are conserved in ORC1 from yeast to mammals.

### Replication foci formation and progression during *P. falciparum* erythrocytic developmental stages in relation to DNA replication

We have already established the correlation between DNA replication and replication foci formation (Fig. S4). It will be interesting to see whether formation and propagation of these nuclear foci during development are related to the rate and timing of DNA replication in the parasites. Several groups have reported the timing and duration of DNA replication in synchronized parasite culture using radioactive nucleotide in the absence or presence of DNA replication inhibitors like hydroxyurea or proteasome inhibitors (Inselburg and Banyal, 1984; Arnot and Gull, 1998; Gantt *et al*., 1998). Based on these results, it is understood that DNA replication initiates after ∼22–24 h following invasion, peaks at ∼30–32 h, decreases thereafter although continuing till 40–44 h. The DNA replication pattern is shown in Fig. S6A based on the results obtained from various studies.

In order to find out whether replication pattern coincides with foci formation pattern, we calculated the number of foci formed at each stage during development for ORC1, ORC5 and PCNA. Number of colocalized foci for ORC5–PCNA and ORC1–ORC5 were also counted. We used acetone-methanol fixation method as it gave us more distinct and slightly higher number of replication foci probably because of the better access of the antibodies to the proteins. Several parasites (more than 100 in each case) from multiple slides were scanned at each developmental stage to rule out the possibility of any bias for the stage of the parasites and number of foci found in each stage. Graphical representation of these results indicates that overall pattern of the foci formation strongly correlates with the DNA replication pattern. Comparison of individual PCNA and ORC5 foci formation reveals that foci formation starts during early-to-mid trophozoite stage for both of them (Fig. S6B). The number of foci further increases with further development, reaching the maximum number during mid-to-late trophozoite stages where DNA replication also peaks. Number of ORC5 foci do not change drastically thereafter whereas the number of PCNA foci drops at the late schizont stage. It is possible that the increase in replication foci per parasite at later stages is due to the increase in genome content or number of nuclei following each round of replication.

Analysis of PCNA–ORC5 merged foci clearly indicates that the formation and progression of such merged foci also follows the DNA replication pattern (Fig. S6C). The number of merged foci peaks during mid-trophozoite stage with a value of ∼15 per parasite, suggesting the presence of these many active replication foci containing both PfPCNA and PfORC proteins during peak replication phase. The numbers decline drastically thereafter with very little association with each other during late schizont stage. ORC1–ORC5 merged foci also follow the same pattern with the maximum number of merged foci visible during mid-trophozoite stages followed by reduction of these numbers at the following stages until late schizont stages where ORC1 foci were completely abolished (Fig. S6D).

It is important to note that the formation, progression and colocalization of PfORC and PfPCNA foci strongly complement biochemical co-immunoprecipitation data to reconfirm our conclusions. The actual number of replication foci per nucleus in the infected parasite may vary as the replication foci number counting is based on the visual inspection of IFA analysis of the different fixed parasites at different time points. Further live cell imaging using transgenic fluorescent parasite line expressing PfORC or PfPCNA fusion protein will be required for quantitative analysis of replication foci formation and progression.

### Perturbation of parasite growth with the intervention of inhibitors affects replication foci formation

**Fig. 6:**
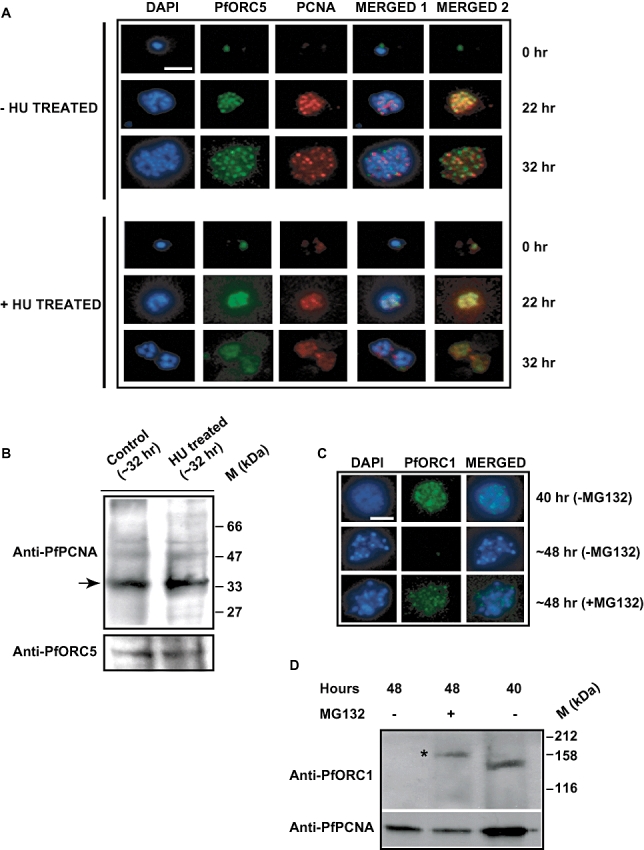
Effects of inhibitors on replication foci formation. A. Effects of hydroxyurea on PfPCNA and PfORC5 foci formation. Synchronized ring stage parasites were grown either in the presence or absence of hyroxyurea (70 μg ml^−1^) and parasites were harvested at different time points (as shown on the right) for immunofluorescence assay using anti-PfORC5 and anti-PfPCNA antibodies followed by respective secondary antibodies. The bar as shown in the inset is equivalent to 3 μm. B. Western blot analysis using anti-PfPCNA and anti-PfORC5 antibodies using parasite pellet before and after drug treatment. C. Effect of proteasome inhibitor MG132 on PfORC1 expression. Synchronized parasite cultures at early-to-mid schizont stages (∼40 h following synchronization) were incubated either in the absence or presence of 100 nM MG132 for 7–8 h until the untreated parasites reach late schizont stage. Glass slides were made using parasites obtained from control and drug-treated culture followed by immunofluorescence assay using anti-PfORC1 antibodies and ALEXA fluor 488 conjugated secondary antibodies. D. Top panel shows the Western blot analysis using anti-ORC1 antibodies against the parasite pellet obtained from the same samples as shown in Fig. 6C. MG132-treated parasites showed the presence of a shifted band (*) at the late schizont stage. The bottom panel shows the Western blot analysis using anti-PfPCNA antibodies as used in the upper panel.

To investigate further whether PfORC and PfPCNA foci are true representation of replication foci, we used DNA replication inhibitor hydroxyurea that blocks the elongation of DNA replication fork by depleting the pool of nucleotides (Thelander and Reichard, 1979). It has been reported earlier that the addition of hydroxyurea (∼60 μg ml^−1^ or more) in synchronized ring stage parasites arrests them in early trophozoite stage corresponding to the time of initiation of DNA synthesis (Inselburg and Banyal, 1984). Accordingly, *P. falciparum* 3D7 parasites were synchronized at the ring stage and parasites were grown either in the absence or presence of hydroxyurea (∼70 μg ml^−1^). Analysis of the morphology of the parasites following Giemsa staining after ∼32 h of drug treatment (Fig. S7A) shows that they are blocked at the trophozoite stage as reported earlier (Inselburg and Banyal, 1984) whereas the untreated parasites grow normally. Immunofluorescence assay using anti-PfORC5 and anti-PfPCNA antibodies show normal progression of foci formation and propagation in the absence of drugs. Incubation of the parasites in the presence of drugs (∼32 h, post treatment) results in reduced and fragmented nuclei compared with the control parasites as shown in DAPI stained parasites (Fig. 6A, panels 3 and 6 respectively). Interestingly, the majority of the parasites (∼85–90%) showed diffused staining pattern for both ORC5 and PCNA following ∼22 h and ∼32 h of drug treatment compared with the distinct foci containing parasites present in the untreated culture. Finally, Western blot analysis using anti-PfPCNA and anti-PfORC5 antibodies against parasite lysate obtained from drug-treated and untreated samples revealed the presence of ORC5 and PCNA both in the absence and presence of drug although they failed to form distinct foci in the majority of the parasites following hydroxyurea (HU) treatment at higher concentration (Fig. 6B).

The mode of action of HU has been reported to be the depletion of the pool of dNTPs by inhibiting ribonucleotide reductase and it should not affect the loading of pre-RC components like ORC. The absence of ORC5 foci formation in the presence of ∼70 μg ml^−1^ HU was a bit surprising to us. Therefore, to address this issue thoroughly, we used different concentration of HU (0, 20, 40 and 70 μg ml^−1^) and followed the formation of replication foci at different time points by immunofluorescence assay (Fig. S8A). The average number of foci per RBC-infected parasite was also calculated (∼100 RBC-infected parasites were counted at each HU concentration at two different time points, 22 h and 60 h post treatment respectively) and plotted accordingly (Fig. S8B). We find that following ∼22 h of drug treatment, increasing concentration of HU affects ORC5 foci formation (and PCNA foci too) gradually with minimum effect at 20 μg ml^−1^ HU concentration, moderate effect at 40 μg ml^−1^ drug concentration and maximum effect at the highest drug concentration (70 μg ml^−1^) (Fig. S8A and B). This effect was more prominent following ∼60 h following drug treatment (the next cycle) where ORC5 foci formation was further decreased (Fig. S8B) even in the lower concentration of drugs (20–40 μg ml^−1^). HU-treated parasites were able to re-invade fresh RBCs at lower concentration (20–40 μg ml^−1^) as evidenced with the increase in parasitemia during second cycle compared with that of 70 μg ml^−1^ HU-treated parasites (data not shown).

It is possible that HU treatment may activate checkpoint in the parasites, leading to the stall of DNA replication, decrease in average number of replication foci and growth arrest as shown in other eukaryotes (reviewed in Nyberg *et al*., 2002). At higher drug concentration (70 μg ml^−1^), it may cause a very drastic and immediate effect on replication foci formation and growth arrest. In order to test the hypothesis whether a checkpoint is activated in HU-treated parasites, we incubated HU-treated parasites in the absence or presence of caffeine. Caffeine can override both S-M and G_2_-M DNA damage checkpoints (Moser *et al*., 2000). We find that addition of caffeine (100 μg ml^−1^) in HU-treated culture (70 μg ml^−1^) increases the ORC5 foci containing parasites two to three times compared with the HU-treated culture only (Fig. S8C). The re-appearance of foci in some parasites was truly reflected in parasitic growth to some extent. The parasitemia of only HU-treated parasites did not alter after 50 h post drug treatment (next cycle) whereas caffeine-treated parasites (in the presence of HU) showed greater than twofold increase in parasitemia (Fig. S8D). These results support our hypothesis of activation of checkpoint in the parasites in the presence of HU, leading to the inhibition of DNA replication and parasitic growth.

To further investigate the mechanism of disappearance of PfORC1 at the late schizont stage, we added different concentration of MG132 (100 nM, 1 μM and 10 μM respectively), an inhibitor of proteasomal degradation pathway after ∼40 h post invasion in the synchronized parasite culture. Analysis of the morphology of the parasites following drug treatment reveal slower growth of the parasites following 100 nM drug treatment compared with the untreated parasites whereas higher concentrations of drugs inhibit parasite growth completely with immediate effect (Fig. S7B). Immunofluorescence assays were performed using anti-PfORC1 antibodies using parasites derived from 100 nM drug-treated and untreated culture after 8 h following the addition of drug. The results indicate that PfORC1 signal can be detected in the untreated parasites before the addition of MG132. However, PfORC1 signal is completely abolished in the same untreated samples following 8 h at the late schizont stage whereas PfORC1 foci can still be visible in the drug-treated parasites, suggesting that MG132 can protect PfORC1 from getting degraded at late schizont stage (Fig. 6C).

Further, we performed Western blot analysis using anti-PfORC1 antibodies against parasite lysate derived from untreated and drug-treated samples. Untreated samples (∼48 h) do not show any band corresponding to ORC1, suggesting that ORC1 is degraded at the late schizont stage (Fig. 6D). Parasite lysate derived from samples before drug treatment (∼40 h) clearly shows a band corresponding to PfORC1. Interestingly, drug-treated samples (∼48 h) shows a band corresponding to ORC1 that is shifted upward compared with the position of ORC1 band in the ‘40 h’ time point lane, suggesting that treatment of MG132 prevents degradation of PfORC1 that might have undergone some post-translational modification during late schizont stage. As a control, Western blot experiments using anti-PfPCNA antibodies show the presence PfPCNA in both in MG132-treated sample and in control sample.

## Discussion

The putative PfORC5 homologue shows ∼20% identity and ∼43% homology with ScORC5 at the C-terminal region. The presence of functional NTP binding domain (hallmark of ORC5 proteins), ability to complement a yeast mutant *ORC5* strain and colocalization of ORC5 with ORC1 and PCNA during DNA replication suggest that it is the best candidate for ORC5 homologue in *P. falciparum*.

Apart from PfORC1 and PfORC5, only a putative homologue of ORC2 has been identified in *P. falciparum* genome, suggesting that either PfORC contains limited number of subunits or it contains functional homologues of other subunits.

**Fig. 7:**
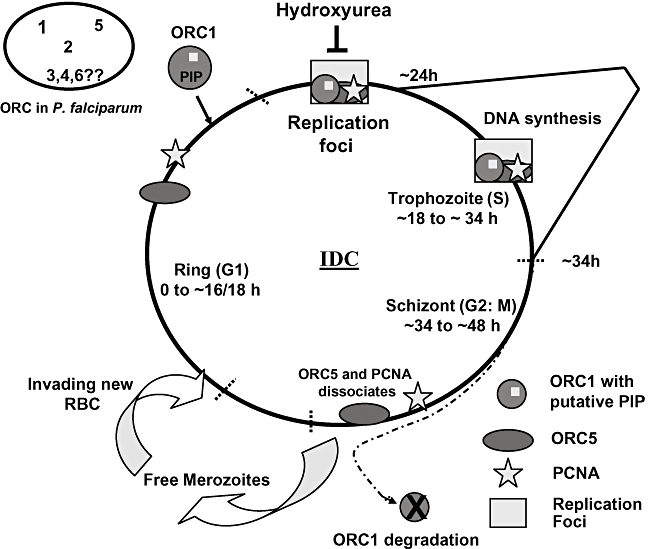
Model of replication foci formation and progression in *P. falciparum*. *P. falciparum* ORC may contain fewer subunits than other eukaryotes with confirmed PfORC1, PfORC5 and a putative PfORC2 subunit. During intraerythrocytic developmental cycle (IDC), different proteins are regulated differently. Based on our immunofluorescence and Western blot data and other published data, PfORC1 is expressed during late ring or early trophozoite stage whereas both PfPCNA1 and PfORC5 are present through all stages. At the onset of DNA replication, during early trophozoite stage, these proteins colocalize with each other and form replication foci. These foci continue to replicate nuclear DNA till late trophozoite/early schizont stage. During schizont stage, these proteins follow different pathways. PfORC5 and PfPCNA slowly detach from each other until late schizont stage where the majority of these foci are separated from each other. However, PfORC1 is completely degraded at this late schizont stage. G1, S and G2/M phases are also indicated according to parasitic developmental stages.

We propose the following model for the regulation of DNA replication in *P. falciparum* (Fig. 7). Following schizogony, the newly released merozoites invade new erythrocytes to initiate the ring stage (G1 phase). PfORC1 is not present at the protein level during early ring stages but gets expressed during late ring/early trophozoite stage parasites (Fig. 4A). ORC5 and PCNA are present at the late ring/early trophozoite stage parasites (Fig. 3A). As the parasites mature further, these proteins form distinct colocalized replication foci. ORC1 with putative PIP motif may facilitate the recruitment of PCNA. These replication foci remain intact during the period of DNA replication (S phase) and slowly the proteins forming the replication foci follow different pathways. ORC1 gets degraded during the late schizont stage parasites, while PCNA and ORC5 separate from each other during the late schizont stage ensuring no further DNA replication at this stage. The post-DNA replication stage that includes chromosome segregation, formation of daughter nuclei and cytokinesis leading to the production of new merozoites is considered to be the G2/M phase. This model proposes co-ordinated processing of clustered replication forks (replication factory model) during S phase progression in the parasites and sheds light into our understanding of complex mechanism of DNA replication initiation and progression in *P. falciparum*.

We have identified putative cyclin-cdk phosphorylation sites (SPTK and TPKK respectively) at the N terminus of PfORC1. These putative cdk-phosphorylation sites might undergo phosphorylation followed by ubiquitination-mediated degradation of PfORC1. This is similar to the fate of human ORC1 that is expressed and targeted to chromatin as cells exit mitosis and pre-replicative complexes are assembled. As the cells enter S phase, HsORC1 probably gets phosphorylated in cyclin A-dependent manner followed by ubiquitination and degradation in a proteasome-mediated pathway (Méndez *et al*., 2002). In hamster cells, ORC1 has been reported to be ubiquitinated following release from the chromatin as the cells enters S phase (Li and DePamphilis, 2002). The post-translational modification of HsORC1 and its subsequent degradation at the late schizont stage clearly suggest that PfORC1 is similarly modified and perform regulatory role in *Plasmodium* DNA replication as described above for other species.

The immunocolocalization and co-immunoprecipitation of ORC components and PCNA during DNA replication, the presence of putative PIP domain in PfORC1 and the *in vitro* pull-down experiments with PfORC1 and PCNA are also important and novel findings. These results clearly suggest that ORC and PCNA are the active components of replication foci. The finding of putative PIP motifs in ScORC1 and HsORC1 homologues may suggest its conserved role during eukaryotic DNA replication. ORC1 with the putative PIP box may facilitate loading of PCNA at the replication fork. Recently, a similar function has been proposed for MCM10 (Das-Bradoo *et al*., 2006). Alternatively, it may be suggested that ORC components are not only the part of the pre-RC, they are also active components of the growing replication fork where the putative PIP domain in ORC1 plays a major role to hold these proteins together along with PCNA. The degradation of PfORC1 at the later stage with the concomitant separation of ORC and PCNA foci strengthens the above hypothesis. The separation of PCNA from ORC binding sites can also be attributed to the differential affinity of PCNA towards different proteins during development. In fact, it has been reported earlier that binding of PCNA to its different partners is modulated by different modifications of PCNA, like ubiquitination and sumoylation that help to attract specific binding partners like translesion polymerases and Srs2 helicase respectively, because of the presence of specific binding motifs in these proteins during bypass DNA replication following DNA lesions (Kannouche *et al*., 2004; Papouli *et al*., 2005).

Presence of PfPCNA during the late schizont stage may be required for genome maintenance processes dealing with stalled replication forks, such as translesion synthesis as reported in mammalian cells (Essers *et al*., 2005; Moldovan *et al*., 2007). Additionally, accumulated PCNA on DNA at late stages might recruit some replication factors like Cdt1 that is ubiquitinated by DDB1-Cul4 ubiquitin ligase following interaction with PCNA, thereby clearing the nucleus from Cdt1-mediated licensing activity (Arias and Walter, 2006).

Presence of PfORC5 at the late stage may ensure its availability for the next cycle of DNA replication. Alternatively, as ORC components have been shown to be essential for silencing Hidden MAT left and Hidden MAT right mating-type loci in *S. cerevisiae*, PfORC5 may follow similar suits in gene silencing particularly in *var* gene silencing that needs to be explored further.

In mammalian cells, the organization of replication factories is dependent on chromatin architecture and structure of chromosomal domains that are dictated by nuclear matrix. The presence of histones, chromatin-modifying enzymes (histone acetylases and deacetylases) and nuclear matrix protein coding genes in *Plasmodium* genome suggests that chromatin structure will also play critical role in deciding the fate of the replication foci.

In mammalian cells, ∼10 000 replication sites with an average 1 mbp of DNA per replication site account for chromosomal DNA replication during S phase with approximately six replicons per replication site (Ma *et al*., 1998). In contrast, we find average ∼15 colocalized (PfORC and PfPCNA) replication foci in *Plasmodium* during the peak of DNA replication. Considering that the *Plasmodium* genome size is ∼24 mbp, each replication foci can account for replication of ∼1.5 mbp DNA, a value close to the other eukaryotes. It is interesting to note that the replication rate is faster in *Plasmodium* than in higher eukaryotes. *Plasmodium* genome content increases from 1 N to 16 to 32 N within a span of 12–16 h, whereas mammalian nuclear DNA replicates only once during the same time period. Therefore, the replication foci in *Plasmodium* must contain higher number of replicons compared with that of mammals. It is possible that origin frequency in *Plasmodium* is much higher than in eukaryotes, accounting for the higher number of replicons per foci. This may also explain the distinct colocalization of PCNA and ORC in *Plasmodium* that is not common in higher eukaryotes.

The gradual disappearance of ORC5 foci in the presence of increasing concentration of HU is very surprising. In general, HU depletes the pool of dNTPs and it should not affect the loading of pre-RC components. We believe that increasing concentration of HU has a direct effect on depleting the dNTP pool that may affect the foci formation at later time points. *P. falciparum* undergoes multiple rounds of DNA replication. Depletion of dNTP pools may affect severely the late rounds of DNA replication. Additionally, the foci that are formed initially may diffuse with time. This may explain the gradual decrease of foci formation with increasing drug concentration. This is in consistence with the finding reported by Santocanale and Diffley (1998) that suggests specific prevention of late-S origins in the presence of HU. Higher concentration of HU (70 μg ml^−1^) may activate a checkpoint immediately that can affect foci formation drastically. Re-appearance of ORC5 foci in some parasites and the increase in parasitemia in the presence of caffeine following HU treatment may suggest that DNA damage checkpoint pathways are active in the parasites. Interestingly, we have found two ORFs in *Plasmodium* database (PF14-0516 and PFB0815w respectively) that show considerable homology with human Chk1 and Chk2 proteins. However, this needs to be verified further.

DNA replication is a fundamental process from bacteria to mammals. However, this important aspect of biology has not been studied thoroughly except the model systems like *Escherichia coli*, *S. cerevisiae* and *Xenopus*. The thorough understanding of this process in protozoan parasites like *Plasmodium* not only highlights the uniqueness of these lower eukaryotes but also confirms the conserved role of the replication proteins among different species. While the presence of previously un-noticed conserved PIP domain in ScORC1, HsORC1 and PfORC1 ensures the conserved role of PCNA in DNA replication among different species, the unique colocalization of ORC and PCNA forming replication foci and their separation following DNA replication clearly highlight important aspect of DNA replication that has not been shown earlier.

Finally, the identification of a critical component of replication machinery in the parasites with nucleotide binding and hydrolysis activities that are central to its function (as found in *S. cerevisiae*) may be useful for ongoing search for finding new targets for intervening *Plasmodium* growth and proliferation.

## Experimental procedures

### Parasite culture

*Plasmodium falciparum* 3D7 strain was cultured in human O^+^ erythrocytes in RPMI 1640 medium supplemented with 25 mM Hepes, 50 mg l^−1^ hypoxanthine, 0.2% NaHCO_3_, 0.5% Albumax (Invitrogen), 0.2% glucose and 10 μg ml^−1^ gentamycin sulphate. Synchronization of cultures was achieved by using 5% sorbitol treatment of cells. Synchronization was verified by morphological analysis of Giemsa-stained infected blood smears at different time points.

For inhibitor studies, hydroxyurea (Sigma) was added into the *P. falciparum* culture (5–6% parasitemia) at different concentrations (0, 20, 40 and 70 μg ml^−1^ respectively) at early ring stage in triplicate. Samples were taken at different time intervals following evaluation of developmental stages of the parasites by Giemsa staining.

The proteasome inhibitor MG132 was added at a concentration of 100 nM in synchronized *P. falciparum* (5–6% parasitemia) culture ∼40 h post erythrocytic invasion in triplicate and followed several hours thereafter.

### RNA extraction and RT-PCR

RT-PCR reactions were performed using cDNA and specific primers for PfORC5, PfMCM4, PfORC1 and control PfGAPDH (Table S1, P15–P22) essentially following the protocol as described elsewhere (Mehra *et al*., 2005). Semi-quantitative analysis of RT-PCR results was performed by densitometric quantification of the amplified products followed by graphical representation of these results.

### ATPase and ATP binding assays

ATPase assay was performed using recombinant pGEX_6P2_-PfORC5Δ1 (171–899 aa) protein (wild type or mutant) following the protocol as described earlier (Mehra *et al*., 2005). Thin-layer chromatography (TLC) was performed to separate the released Pi. The TLC plates were dried, autoradiographed and quantified by Phosphorimager (Fujifilm-BAS-1800).

For ATP binding assay, 1 μg of MBP-PfORC5Δ2 (171–421 aa) protein (wild type or mutant) was cross-linked with [α-^32^P]-dATP in the presence of UV (254 nm) for 30 min in 30 μl reaction mixture containing 20 mM Hepes (pH 7.5), 10% glycerol, 0.1 mM DTT, 10 μCi [α-^32^P]-dATP (3000 Ci mmol^−1^) using a Stratagene cross-linker followed by addition of 0.8 ml of 100 mM dATP and 20 μg of BSA. Proteins were precipitated by trichloroacetic acid and the pellet was washed once with acetone containing 0.5% HCl and twice with acetone. Proteins were separated by SDS-PAGE and the radiolabelled dATP-bound proteins were visualized using phosphorimager.

### Immunofluorescence assay

Thin smear of synchronous culture of infected erythrocytes was made on glass slides. Slides were air-dried for 2 h at room temperature followed by fixation for 30 min in freshly prepared solution of acetone and methanol (90% and 10% respectively) at −20°C. The slides were further blocked for 1 h at room temperature in 1× PBS containing 3% BSA and 0.01% saponin. Slides were then incubated overnight in 1× PBS containing respective primary antibodies at 4°C. Immunoaffinity-purified anti-PfORC1 (rabbit), anti-PfORC5 (rabbit), anti-PfORC1 (mouse) and anti-PfPCNA1 (mouse) antibodies were used at 1:500, 1:500, 1:2000 and 1:500 dilutions respectively, as primary antibodies. Slides were further washed in 1× PBS and incubated for 45 min at 4°C in 1× PBS containing anti-rabbit IgG-Alexa flour 488 or antimouse IgG Alexa fluor 594 (Molecular Probes) 1:1000 secondary antibodies and nuclear stain DAPI. Images were captured using Nikon Eclipse 80i fluorescent microscope.

Alternatively, glass slides containing parasites were incubated in 2% PFA solution for 15 min at the room temperature followed by blocking them in 0.5% BSA solution in 1× PBS for 20 min. The slides were further treated with primary antibodies for 2–3 h at room temperature and secondary antibodies for 45 min with thorough washing with 1× PBS after each antibody treatment. Antibody dilutions were kept the same as above.

### Immunoelectron microscopy

Immunoelectron microscopy for the subcellular localization of PfORC5 was performed as described elsewhere (Mehra *et al*., 2005) using anti-PfORC5 antibodies (1:500) as primary antibodies and goat anti-rabbit IgG secondary antibodies coupled with 10–20 nm gold particles at 1:100 dilution. Sections were viewed using a Morgagni-268 transmission electron microscope.

### Immunoprecipitation assay

For Immunoprecipitation, lysate from 100 μl of *P. falciparum*-infected erythrocyte pellet (8–10% parasitemia) was prepared by adding five to six pellet volume of lysis buffer containing 300 mM NaCl, 20 mM Tris.Cl, pH 8.0 and 0.5% NP-40 and anti-protease cocktail (Sigma). Lysate was clarified by centrifugation at 15 000 *g* for 30 min at 4°C. Either pre-immune or immune sera (1–2 μl) were added to this cleared lysate and the mixture was incubated at 4°C for 2 h with constant mild shaking followed by addition of 20 μl of protein A sepharose slurry or protein G sepharose slurry (Sigma). Finally, these bead Ab protein mixtures were incubated for 1 h at 4°C. Beads were collected and washed three times with 1× PBS containing 0.1% BSA. Immunoprecipitated proteins were eluted from beads by adding 2 vols of SDS-PAGE loading buffer and boiling at 95°C for 10 min. Proteins were separated by SDS-PAGE and transferred onto PVDF membrane and immunoblotted using corresponding primary and secondary antibodies.

### Complementation of yeast ORC5 mutant

Complementation experiments were performed in *S. cerevisiae* using either wild-type PfORC5 or *S. cerevisiae* ORC5 or different chimeras of *S. cerevisiae* and *P. falciparum* ORC5. Details of the methods are described in supplementary data, *Experimental procedures* (yeast complementation section).

### DNA manipulation

The putative *PfORC5* gene was amplified by polymerase chain reaction using *P. falciparum* 3D7 genomic DNA as a template and specific primers (P1 and P2, Table S1). For the detailed cloning strategy of wild-type and mutant forms of PfORC5 and PfPCNA, please see supplementary information.

### Recombinant protein purification

For the purification of PfORC5 fusion recombinant proteins to perform ATP binding and hydrolysis assay, *E. coli* BL21 codon-plus strain was transformed with respective recombinant clones. Of all the constructs made to express recombinant proteins, only pGEX_6P2_-PfORC5Δ1 (171–899 aa) and pMAL_c2×_-PfORC5Δ2 (171–421 aa) yielded the recombinant proteins in soluble form. The detailed methods for protein purification for these constructs and other constructs for antibodies preparation (PfORC5C1 and PfORC5C2) are described in supplementary information, materials and methods.

### Antibody production and Western blotting analysis

Polyclonal antibodies against different antigens in rabbit and mice were generated essentially following the protocol described by Harlow and Lane (1988). Please see supplementary information for detailed protocols.

Western blot analysis was carried out following the standard protocol (Sambrook *et al*., 1989). Please see supplementary information for the details.

## Supplementary material

This material is available as part of the online article from: http://www.blackwell-synergy.com/doi/abs/10.1111/

j.1365-2958.2008.06316.x

(This link will take you to the article abstract).
