# Supplementary material for: Plasmodium falciparum origin recognition complex subunit 5: functional characterization and role in DNA replication foci formation
Source: Mol Microbiol. 2008 Jun 19;69(3):646–65. doi: 10.1111/j.1365-2958.2008.06316.x (PMC2610387; doi:10.1111/j.1365-2958.2008.06316.x)
Supplement: Supplementary file 1 [file mmi0069-0646-SD1.pdf]

Manuscript MMI-2008-07643

"*Plasmodium falciparum* origin recognition complex subunit 5 (PfORC5): functional characterization and role in DNA replication foci formation" by Gupta A *et al.*

Supplementary data: Materials and methods

Complementation of yeast *ORC5* mutant strain. For the complementation assay, a yeast ORC5 swapper strain *YSPB5.1* (a kind gift from Steven P. Bell, MIT, USA) with a deletion of chromosomal copy of *ScORC5* but maintaining the wild type copy in a plasmid with *URA* marker was used. *PfORC5* or *ScORC5* genes were amplified by PCR using *P. falciparum* or *S. cerevisiae* genomic DNA and specific primers (P27-P28 and P23-P24, respectively) as shown in table I followed by cloning them in pRS416 yeast expression vector under *GAL* promoter. Finally, this cassette containing the promoter and the respective genes were taken out using SacI and Acc65I restriction enzymes and ligated into another yeast vector pRS314 having tryptophan as a selection marker. All constructs were confirmed by sequencing. To create ORC5 chimera (with N-terminus of *ScORC5* and C-terminus of *PfORC5*), we used PCR-based SOE (gene splicing by overlap extension) method where two fragments could be joined without introducing any restriction site at the junction (Horton *et al.*, 1989). While deciding the exact regions N-terminus of ScORC5 and C-terminus homology region of PfORC5, we considered the secondary structure of both the proteins so that helix and beta sheet region of individual protein is not disturbed. We also looked for few identical overlapping residues to facilitate the overlapping PCR without introducing any restriction site. This allowed us to fuse ScORC5ΔC (1-184) and PfORC5ΔN2 (561-899) to get Chimera ORC5 (ScORC5ΔC + PfORC5ΔN2). The details are discussed in the result section and they are shown in schematic diagrams (fig. 3A-B). Specific primers were used for the amplification of N-terminus and C-terminus of *ScORC5* and *PfORC5* respectively (P25-P26 and P29-P30 respectively). Chimera specific primers are also shown in table I (P31-P32). Yeast transformation was carried out by lithium acetate method (Gietz *et al.*, 1992). All transformed constructs were plated on fully supplemented without Trp synthetic drop

(SD) out media and plates were incubated at 30^0C for 3-4 days. To test the ability of these constructs to rescue the mutant cells, single colonies were grown in –Trp SD medium at 30^0C and serial dilution test was done simultaneously on -Trp and FOA (1%) containing plates having raffinose and galactose. Plates were incubated at 30^0C for 3-4 days and then images were taken.

DNA manipulation. Different domains of PfORC5 were amplified using specific primers (for PfORC5C1, P7-P8; for PfORC5C2, P9-P10). PCR amplified coding regions were cloned in pET28a vector (Novagen) at EcoRI site. All clones were sequenced subsequently. For GST-PfORC5Δ1 and MBP-PfORC5Δ1 fusion proteins specific forward primer sets P3-P4 were used, while to amplify PfORC5Δ2 domain, P5-P6 primer sets were used. The open reading frames and were subsequently cloned in pGEX6p2 (Amersham Pharmacia Biotech) and pMALc2x (New England Biolabs) at EcoRI site. All primer sequences are shown in Table 1.

To create a point mutation (lysine, AAA to Alanine, GCA) in Walker A nucleotide-binding domain of PfORC5, PCR was carried out using mutagenic oligos (P11-P12) as shown in table1, where pGEX-PfORC5Δ1 or MBP-PfORC5Δ2 were used as templates. Full length PCNA1 ORF was amplified by similar method using P13-P14 primer sets and amplified product was cloned in pGEX6p2 vector in BamHI site. For the cloning of PfORC1C and PfORC1C1 (as shown in fig. 6B and C), respective DNA fragments were amplified using primer sets P33-P34 and P35-P36 respectively followed by cloning them in pMALc2x using EcoRI site. All the recombinant clones were sequenced subsequently.

Recombinant Protein Purification. For protein purification**,** the bacterial culture was grown at 37^0C till the O.D. reached 0.4-0.6 and then induced for expression of recombinant proteins using 1 mM IPTG. For the purification of recombinant proteins having His_6-tag, cells were harvested from 4 liter bacterial culture and resuspended in 30 ml lysis buffer (100 mM Na_2HPO_4, 100 mM NaH_2PO_4, 50mM Tris-Cl. pH-8.0, 300 mM NaCl, 10 mM β mercaptoethanol and 100 μM PMSF). Truncated His_6-PfORC5C1 and His_6-PfORC5C2 recombinant proteins were purified by solubilizing them in 1% lauroyl sarcosine and purified by Ni-NTA (Qiagen) affinity chromatography following the similar protocol as described elsewhere (Mehra *et al.*, 2005).

For purification of MBP tagged recombinant proteins (MBP-PfORC5Δ2), following induction at 22^{0}C for 8 hr, bacterial cells were harvested from 1 litre culture and resuspended in 20 ml lysis buffer (20mM Tris-Cl, pH 7.4; 300mM NaCl, 1mM EDTA, 10 mM β mercaptoethanol and 100 μM PMSF). The protein was further purified by affinity binding to amylose resin (NEB) followed by washing once with 1M NaCl to remove any contaminating protein and twice by lysis buffer as described elsewhere (Mehra *et al.*, 2005). MBP-Fusion protein was eluted by buffer containing 10 mM maltose. MBP protein was also purified in similar fashion.

GST-PfORC5Δ1 recombinant protein was also induced in similar manner and cells were harvested from 2 litre culture and resuspended in 20 ml lysis buffer (1xPBS, 2mMEDTA, 5mMDTT and 100μM PMSF and Protease inhibitor cocktail). Soluble protein fraction (after centrifugation at 10,000xg for 15 min) was incubated with Glutathione-sepharose-4B beads for 1 hr at 4^{0}C followed by washing the beads three times in the presence of lysis buffer, before eluting the proteins with elution buffer containing 20 mM reduced Glutathione. Same protocol was followed for the purification for GST protein. Recombinant GST-PfPCNA was also purified in a similar fashion. All the proteins were dialysed, aliquoted and stored at -80^{0}C.

Antibody production and Western Blotting analysis. To generate the polyclonal antibodies against His_6-PfORC5C1 and His_6-PfORC5C2, purified proteins were run on SDS-PAGE and respective bands were excised from the gel. These gel slices containing the desired protein were chopped, crushed and resuspended in 1x PBS to make fine paste and mixed with Freund's adjuvant. Antigenic mixture containing His_6-PfORC5C1 was used to immunize two rabbits while His_6-PfORC5C2 was used as antigen for the immunization of mice. Antibodies form the final bleed were affinity purified against corresponding recombinant proteins by immobilizing them onto nitrocellulose membrane. Similar protocols were used to generate antisera against PfPCNA protein in mice. Western blot analysis was carried out following the standard protocol (Harlow and Lane, 1988) against recombinant proteins to test the titre and specificity of generated polyclonal antibodies.

To investigate the expression pattern of PfORC5 and PfPCNA protein in different *P. falciparum* developmental stages, synchronized parasite cultures were treated with 0.1% saponin to release the parasites. Released parasites were washed with 1x PBS and centrifuged at 3000xg for 5 min. Parasite pellets were mixed with 2x Lemelli buffer and boiled for 10 min at 95^0C and immediately loaded on 10% SDS-PAGE to resolve the proteins. These proteins were transferred on PVDF membrane (Millipore) and western blot analysis was performed as per standard protocol using antibodies against PfORC5 or PfPCNA or PfORC1.

Supplementary data:

Table I: List of Primers:

**Primers**	**Primer sequence**
PfORC5Fw **(P1)**	5'-CGGAATTCATGTATAAAATAAAAAATAACGAG-3'
PfORC5Rv **(P2)**	5'-CGGAATTCTTATATTATCAACTCATCTAGAGG-3'
PfORC5N1Fw **(P3)**	5'-CGGAATTCGATATCTATAAAGAAGCAAAAAAATG-3'
PfORC5N1Rv **(P4)**	-------------- Same as PfORC5Rv ---------------
PfORC5N2Fw **(P5)**	--------------- Same as PfORC5N1Fw -------------
PfORC5N2Rv **(P6)**	5'-CGGAATTCTCAATTAGAAGGGCTCCTAATATTTTC-3'
PfORC5C1Fw **(P7)**	5'-CGGAATTCGATAATATAAGATATTTAGTTAGG-3'
PfORC5C1Rv **(P8)**	--------------- Same as PfORC5Rv ----------------
PfORC5C2Fw **(P9)**	5'-GGAATTCCATATGTGGCCATTATTTATTAAACC-3'
PfORC5C2Rv **(P10)**	---------------- Same as PfORC5Rv ---------------
ORC5MutFw **(P11)**	5'-GGTTTACCTGGTATGGGAGCACAAAAGTAG-3'
ORC5MutRv **(P12)**	5'-CTACTTTTGTTGCTCCCATACCAGGTAAACC-3'
PfPCNAFw **(P13)**	5'-CGGGATCCATGTTAGAGGCCAAATTAAATAAT-3'

PfPCNARv **(P14)** 5'-CGGGATCCTTAATCTTTATTATCCATATCGTC-3'

ORC5RTFw **(P15)** 5'-AGTTTAATATGTCAACAAATTAATAC-3'

ORC5RTRv **(P16)** 5'-TTATATTATCAACTCATCTAGAGG-3'

MCMRTFw **(P17)** 5'-AGGAGCAGTCGTATTATCAGATAAGG-3'

MCMRTRv **(P18)** 5'-TTTCTAGCTCTCTGTTGTGTAATATAG-3'

ORC1RTFw **(P19)** 5'-GTACTCATTTTCTATACACCT-3'

ORC1RTRv **(P20)** 5'-AAAGATTTATTTTTATTTAAC-3'

GAPDHRTFw **(P21)** 5'-ATGCCAAGTAGATGTTGTATG-3'

GAPDHRTRv **(P22)** 5'-TCGTACCATGAAACTAATTTG-3'

ScORC5Fw **(P23)** 5'-CGGGATCCATGAATGTGACCACTCCGGAAG-3'

ScORC5Rv **(P24)** 5'-CGGGATCCTCATTCGTGAATATCGCTCAAG-3'

ScORC5NFw **(P25)** ---------------- Same as ScORC5Fw ----------

ScORC5NRv **(P26)** 5'-CGGAATTCTCAAATGCAATGTGTAGAATATCTTTG-3'

PfORC5YFw **(P27)** 5'-CGGAATTCATGTATAAAATAAAAAATAACG-3'

PfORC5Yrv **(P28)** 5'-CGGAATTCTTATATTATCAACTCATCTAGAG-3'

PfORC5YCFw **(P29)** 5'-CGGAATTCATGCCAACTGTTTGGTTTGATTC-3'

PfORC5YCRv **(P30)** ---------------- Same as PfORC5Rv ----------------

ChimeraRev **(P31)** 5'-CCAAACAGTTGGAATGCAATGTGTAGAATATC-3'

ChimeraFw **(P32)** 5'-ACACATTGCATTCCAACTGTTTGGTTTGATTCA-3'

PfORC1C Fw (**P33**) 5'-CGGGATCCAAGGAATATATGAATAAAGCTCAAA-3'

PfORC1C Rv (**P34**) 5'-CGGGATCCCTAGTAAAAGTTTAATTTCTTCG-3'

PfORC1TrFw (**P35**) 5'-CGGGATCCAAGGAATATATGAATAAAGCTCAAA-3'

PfORC1TrRv (**P36**) 5'-CCCAAGCTTTCATATGGATACATCTCTGTTATC-3'

Supplementary data: Figure Legends:

Supplementary figure 1. Primary sequence analysis of putative PfORC5 homolog. The amino acid sequences of PfORC5 and ScORC5 are aligned using CLUSTALW program. The repeat regions I and II contain asparagine, aspartic acid and lysine rich residues as marked by straight line on top. The putative nuclear localization signal (NLS) and conserved NTP-Binding motifs are also shown. The box shows the homology region between the two proteins at the C-terminal region.

Supplementary figure 2. A. PCR amplification of *PfORC5* gene from 3D7 genomic DNA using specific primers as shown in table I. Control lane shows no amplification product in the absence of genomic DNA. 'M' stands for DNA ladder marker in kb. **B.** Schematic diagram of PfORC5 showing the NTP-binding domain, C-terminal ORC5 homology domain and repeat regions (I and II). Solid lines below indicate the different deletion mutant proteins used for raising polyclonal antibodies in rabbit and mouse respectively. Arrowheads indicate forward and reverse primers (P15-P16) used for RTPCR analysis**. C.** Western blot analysis to analyze the expression of PfORC5 at the protein level using either mouse anti-PfORC5 or pre-immune sera. Molecular mass markers are shown at the right.

Supplementary fig. 3. A. Immunofluorescence assay to show the expression pattern of PfORC5 (green) and PfPCNA (red) during the late schizont stage. The extreme panel shows the DAPI stain of the nuclei. Merged and merged (enlarged) panels show that PfORC5 and PfPCNA do not co-localize with each other during late schizont stage in contrast to trophozoite stage parasites. The bar is equivalent to 3 micron. **B.** Immunprecipitation assay. Parasite extract from the mixed stage parasites was used for immunoprecipitation (IP) using either anti-PfPCNA or pre-immune sera (top panel) followed by immunoblot (IB) using anti-PfPCNA antibodies . Input lane shows the presence of PCNA in the extract. '*' indicates the light chain of the immunoglobulins.

Second panel shows the immunoprecipitation of PfORC5. Arrowheads indicate the positions of the respective proteins.

Supplementary fig. 4. Correlation of parasite stage progression with DNA content. A. Parasites were synchronized tightly and harvested at different time points as mentioned above followed by giemsa staining to confirm their growth stages. **B.** Total genomic DNA was extracted from each sample and they were resolved by agarose gel electrophoresis. **C.** The DNA from above samples was quantified spectrophotometrically and plotted accordingly. The DNA content increased significantly as the parasites progressed from ring to schizont stages.

Supplementary fig. 5. Immunolocalization of PfORC5 and PfPCNA during intra erythrocytic developmental stages. Parasites harvested from different stages as shown on the left were harvested and fixed using paraformaldehyde (PFA) and processed for immunofluorescence using anti-PfORC5 and anti-PfPCNA antibodies as described in the materials and methods. Merged 1 panel shows the merged images of green PfORC5 and red PfPCNA signals whereas merged 2 panel shows the merged images of nuclear DAPI, green PfORC5 staining and red PfPCNA staining only. The bar on the left corner top panel is equivalent to 3 micron.

Supplementary fig. 6. Co-relation of replication foci formation and progression with DNA replication pattern in *P. falciparum.* A. DNA replication pattern during erythrocytic developmental stages. Based on the radio-nucleotide uptake studies published earlier, the pattern of DNA replication in synchronized parasite culture is represented graphically as percent DNA synthesis at different time points. Duration of ring, trophozoite and schizont stages is also indicated. **B.** Replication foci dynamics during parasite development. PfPCNA and PfORC5 foci were calculated at different erythrocytic developmental stages as indicated at the bottom. At least twenty-five parasite nuclei were scanned from the same stage and the average number of foci was calculated and plotted in each case. **C.** Comparison of PfPCNA foci with PfPCNA and PfORC5 merged foci during development. The foci were calculated at the different developmental

stages from at least twenty-five nuclei at each stage and the average values were plotted graphically. **D.** Comparison of ORC5, ORC1 and PfORC5-PfORC1 merged foci during development.

Supplementary fig. 7. A. Giemsa staining of *P. falciparum* with or without hydroxyurea (HU) treatment. Synchronized parasites were treated with HU (70 µg/ml) at ring stage. Samples were collected at different time points from HU untreated and treated parasites. Giemsa staining was performed to check the effect of drug on parasite growth. **B.** Giemsa staining of MG132 treated and untreated samples. Synchronized parasites (~40 hours post-synchronization, '0' hr) were treated with different concentrations of MG132, as indicated on the right. Samples were taken out at different time points (as indicated on top) to check the morphological changes by Giemsa staining.

Supplementary fig. 8. A. Effect of HU on foci formation. Immunofluorescence assay to show the formation of PfORC5 (green) and PfPCNA (red) foci in the absence and presence of different concentration of HU (as indicated on the right) following ~22 hrs drug-treatment. **B.** Average number of ORC5 foci per infected RBC. At least 100 parasite infected RBC were selected for ORC5 foci counting and average number of foci were calculated at two different time points ('22' hrs and '60' hrs, 1st and 2nd cycle respectively) for each drug concentration (0-70 µg/ml). **C.** Caffeine enhances the number of parasites with ORC5 foci even in the presence of HU. ORC5 foci containing parasites were counted either in the presence of HU (70 µg/ml) alone or HU (70 µg/ml) and caffeine (100 µg/ml) together. **D.** Parasitemia were calculated at two different time points ('0' hr and '50' hr post-drug treatment, 1st and 2nd cycle respectively) either in the presence of HU (70 µg/ml) alone or HU (70 µg/ml) and caffeine (100 µg/ml) together. The same were plotted to compare the effect of caffeine to override the effect of HU.

```
                                                       Putative NLS (56-72)
ScORC5    ------------------------------------------------------------
PfORC5    MYKIKNNESDINSDDCNETAQECIYGFSSPKKSRKESPIFVQNNDDTCSSNNIYEKKTCS 60

                                   Repeat region I (81-170)
ScORC5    ------------------------------------------------------------
PfORC5    NTSASSVKKYDKKEQSLCSDINNYNKVNIEGLKINERNYDRINNSEEETNINDDNNDDNN 120

ScORC5    ------------------------------------------------------------
PfORC5    GDYDDDNNSDDDDDNDDNNNNDDNNNDDDEDVDDFEDIKENDEYKDPTYSDIYKEAKKCN 180

ScORC5    ------------------MNVTTPEVAFREYQTNCLASYISADPDITPSN---------- 32
PfORC5    IRCENIMNSSVNKKNLEEINESDPLNSSDNSMTSSSEESCSEESDKESDKESDKDGNLYD 240
                            :* : *  :  :  *..  .  * :.*  ..:

ScORC5    ----------------------------------------------------------LI 34
PfORC5    EELNEIIEEGYFKELIPAIPHDILQAYISCLKICGFERQVQLHRLINLLGDLRDPISVIQ 300
                                                                    :
          NTP-Binding Domain
ScORC5    LQGYSGTGKTYTLKKYFNAN-------PNLHAVWLEP--VELVSWKPLLQAIAR--TVQY 83
PfORC5    ILGLPGMGKTKVVKNFIKLTNVPFAYVNCLMAVYQSGRSAKNVIYHTILKDLSINLLNEF 360
          : * .* *** .:*:::: .         * **: .   .: * ::.:*: ::     ::

ScORC5    KLKTLYPNIPTTDYDP-----------------------LQVEEPFLLVKTLHNIFVQYE 120
PfORC5    NEYKKINNITNYSYDPTKLVPNHVSNTDNFFSILHKLLSFKPEDILNNKRTTENIRSPSN 420
          :  .   **.. .***                       :: *: :   :* .**    :
                          Repeat region I I (422-480)
ScORC5    SLQEKT------------------------------------------------------ 126
PfORC5    SNNNKKKKKEQNDSTGKNSKEECNNNEDDDDDNNKNNFNNNNSNNVRFNSNTNYYKDKLY 480
          * ::*.

ScORC5    --CLFLILDGFDSLQDLDAALFNKYIKLNELLP-------KDSKINIKFIYTMLETS--- 174
PfORC5    DRSVVFILDNIRYLVRTHPDLFYALTRIHEYIKGPYNDVTKANKTTRGLCIILINRSPLP 540
            .:.:***.:  *    ..**    :::* :        * .* .  :   ::: *

ScORC5    -------------FLQRYSTHCIPTVMFPRYNVDEVSTILVMSRCGELMEDSCLRKR--I 219
PfORC5    DEIFDGLPQPPTVWFDSYTSEMCKNILYRLYNSMCFESLLTYNDKDLKIYYVKHNKNEFL 600
                       ::: *::.   .:::  **   ..::*. .  .  :     .*.  :

ScORC5    IEEQITDCTDDQFQNVAANFIHLIVQAFHSYTGNDIFALNDLIDFKWPKYVSRITKENIF 279
PfORC5    IKRNDVILENDVIYDIWCRYVDYIINVSYKDYKSDFHELLFICSHMWPLFIKPILDG-VL 659
          *:.: .   :* : :: ..::. *::. :.   .*:. *  : .. ** ::. * .  ::

ScORC5    EPLALYKSAIKLFLSTDDNLSENGQG-------ESAITTNRDDLENSQTYDLSIISKYLL 332
PfORC5    EPIVENMNALQRNIDTHIRVATYNHSSHFTFELIDSVFLNENNLKN--KIDLSFYSKILL 717
          **:.   .*::  :.*. .::  .:.        .::  *.::*:*  . ***: ** **

ScORC5    IASYICSYLEPRYDASIFSRKTR-----IIQGRAAYGRRKKKEVNPRYLQPSLFAIERLL 387
PfORC5    VGAYLASRNLPLTDKRFFNATVKGGAFTLPKKRKGKNKNESILTLLSKSIPKNFTFIRWL 777
          :.:*:.*   *  *  :*. ..:     : : * . .:.:.  .      *. *:: * *

ScORC5    AIFQAIFP------------------------IQGKAESGSLSALREESLMK-------- 415
PfORC5    CLTDCLLVCFFDEQLILNSLICQQINTLIQLGFISFSSPNNLSCLVRNSLMNGVQWSGYC 837
          .: :.::                         : . :....**.* .:***:

ScORC5    --ANIEVFQNLSELHTLKLIATTMNKNIDYLSPKVRWKVNVPWEIIKEISESVHFNISDY 473
PfORC5    GSALLNTTTNFSSLTNNIFCETNNSMTYESLDPYTKLVIQVPEETIRNISKEMKIPLDEL 897
            * ::.  *:*.* .  :  *. . . : *.* .:  ::** * *::**:.::: :.:

ScORC5    FSDIHE 479
PfORC5    II---- 899
          :
```

C-terminal ORC5 homology region

Supplementary fig. 1 *, identical; :, strongly similar; ., weakly similar

A.

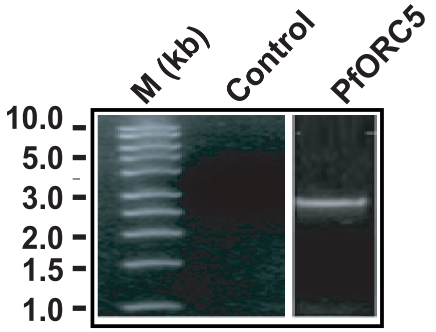

B.

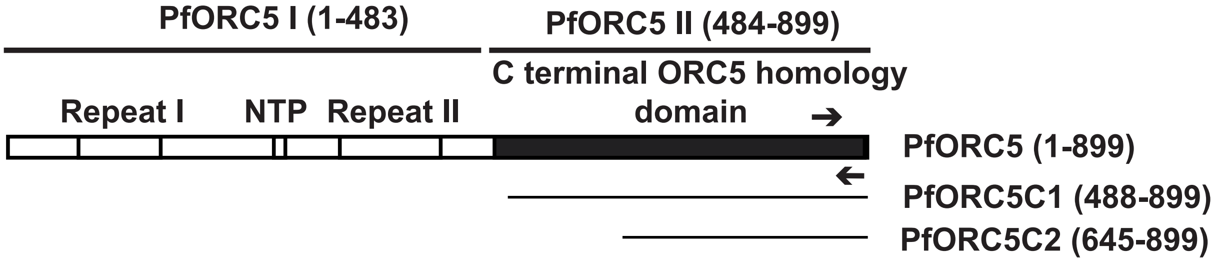

C.

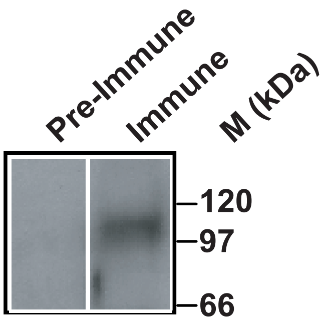

Supplementary fig. 2

A.

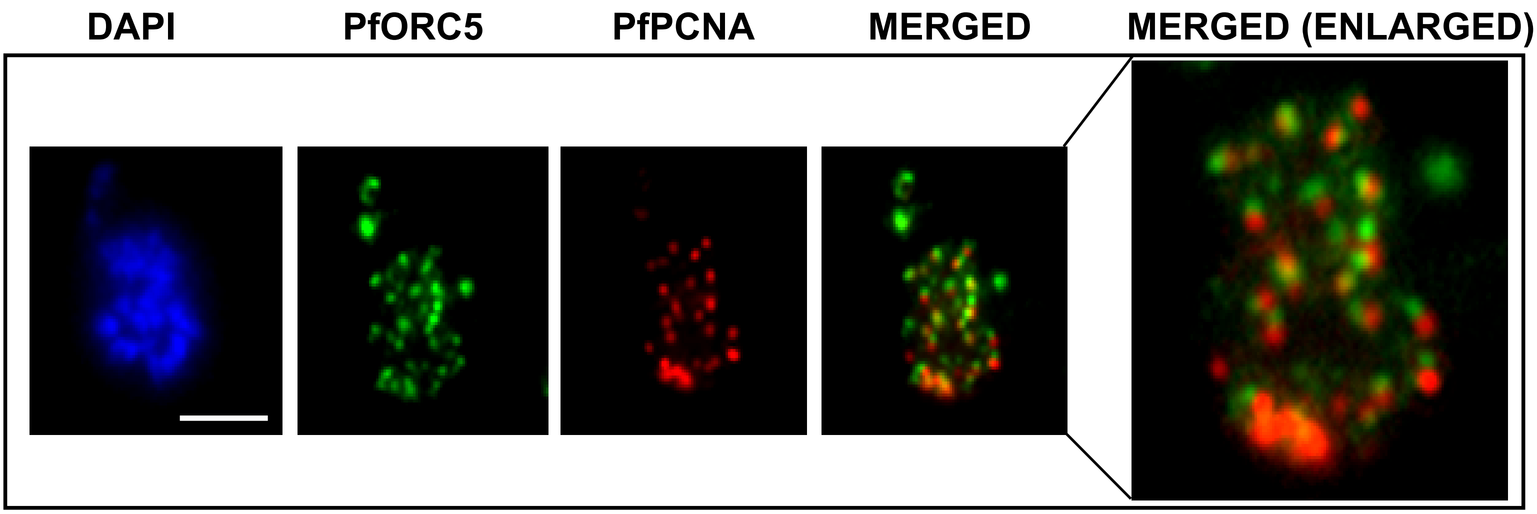

B.

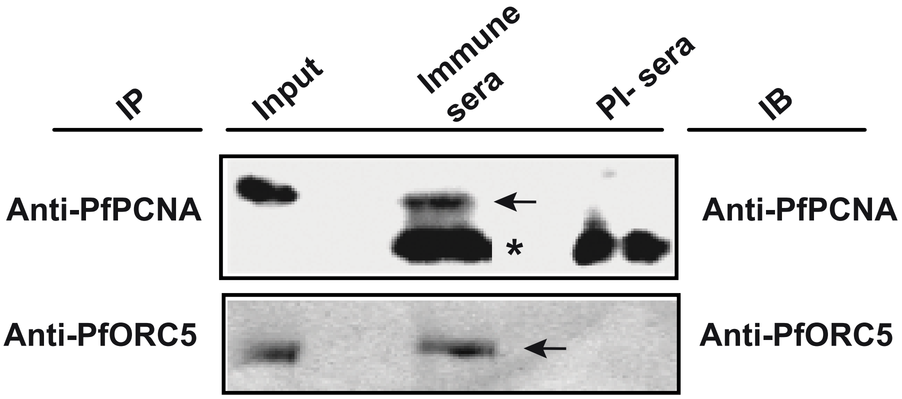

'*' indicates light chain of immunoglobulinns

Supplementary Fig. 3

A. Synchronized parasites from different developmental stages

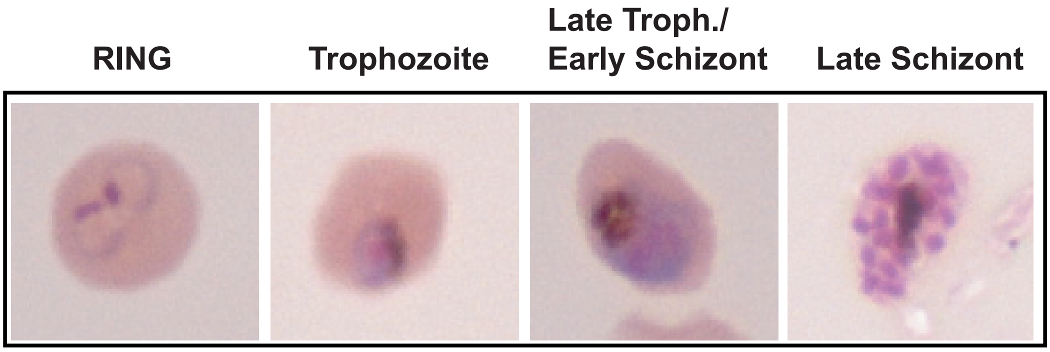

B. Genomic DNA resolved in agarose gel from the above stages

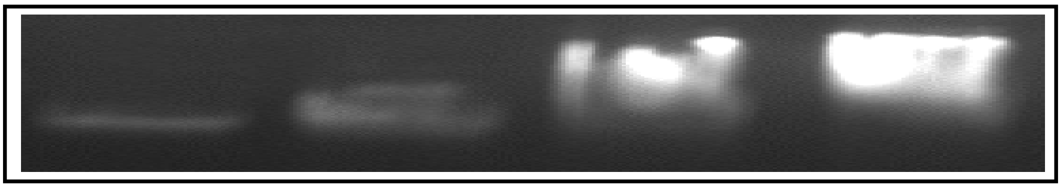

C. Quantitation of Genomic DNA obtained from the above stages

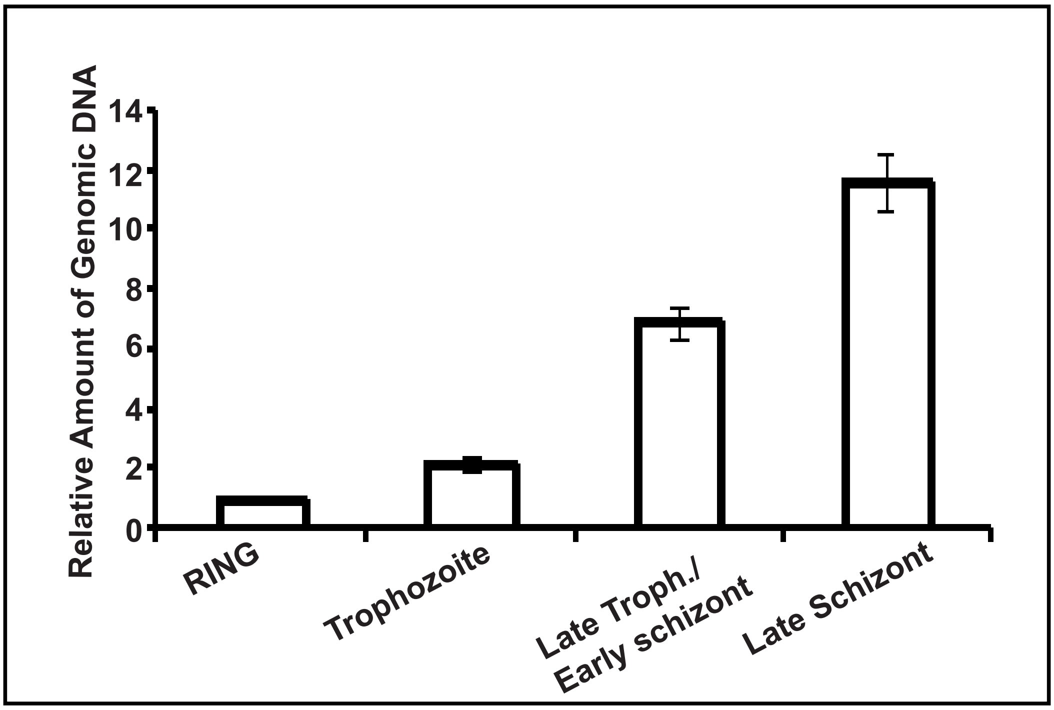

Supplementary Figure 4.

IMMUNOLOCALIZATION OF PfORC5 AND PfPCNA DURING INTRA-ERYTHROCYTIC DEVELOPMENTAL STAGES

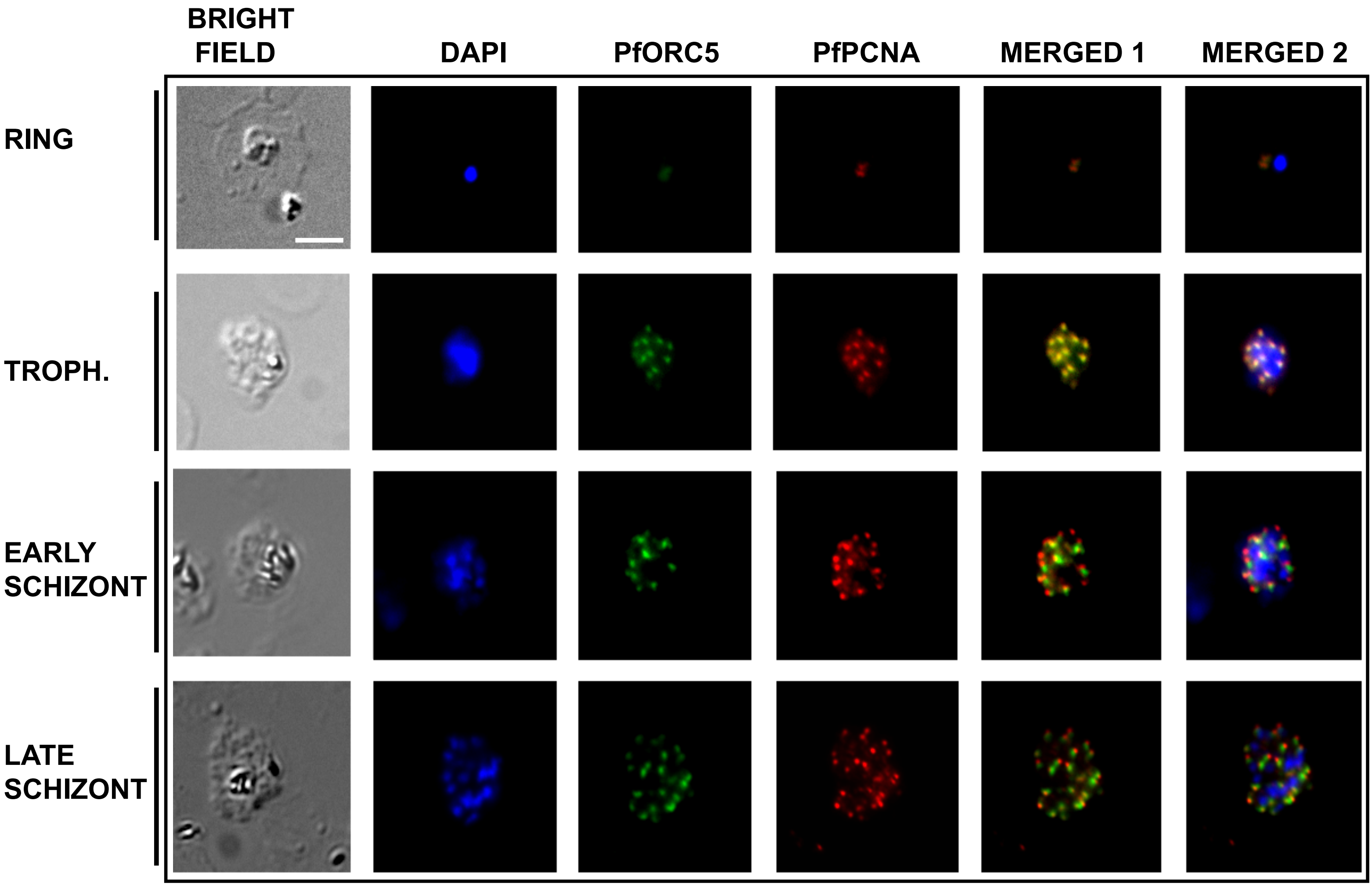

SUPPLEMENTARY FIGURE 5

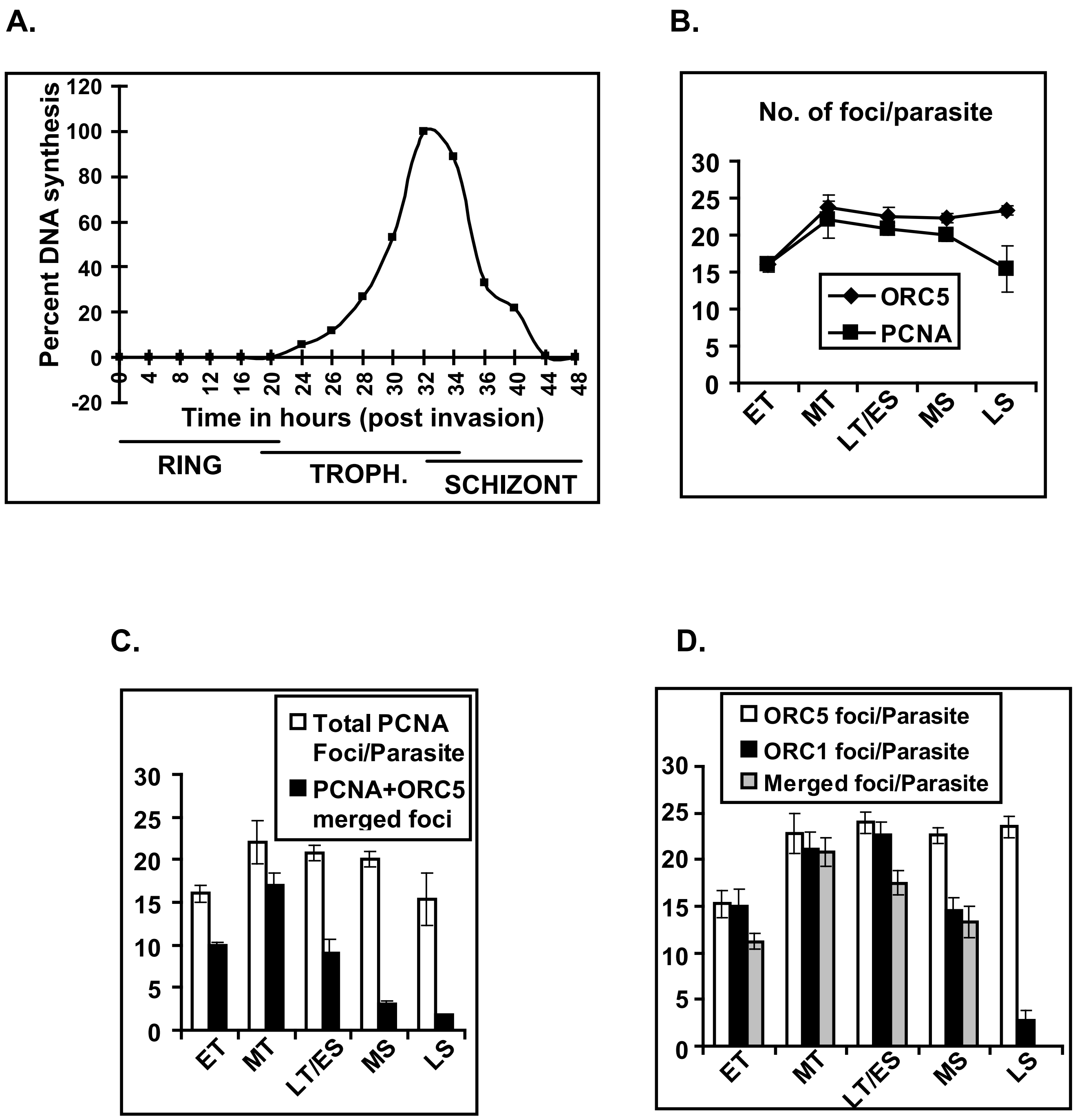

Supplementary fig. 6

A.

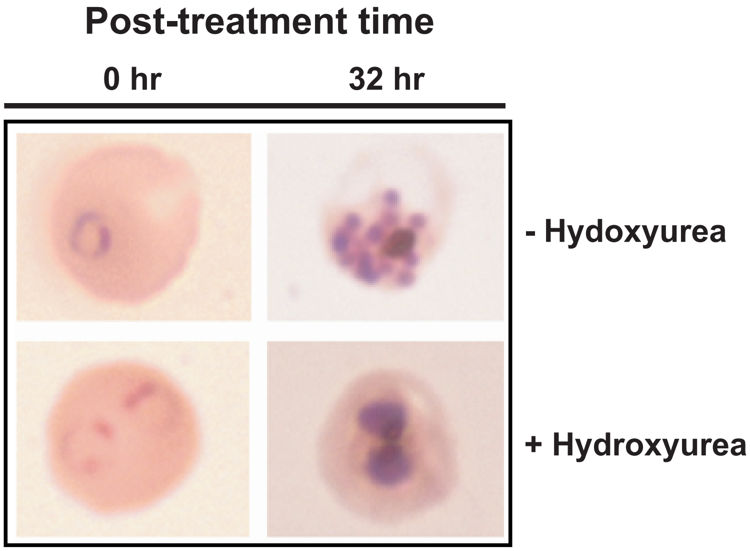

B.

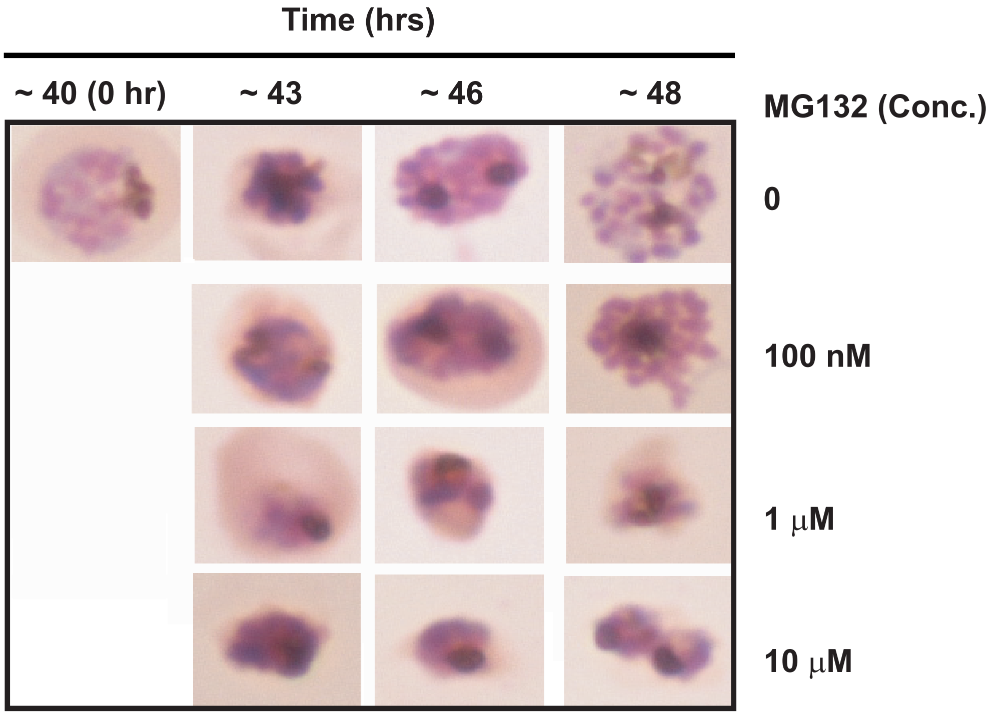

Supplementary figure 7

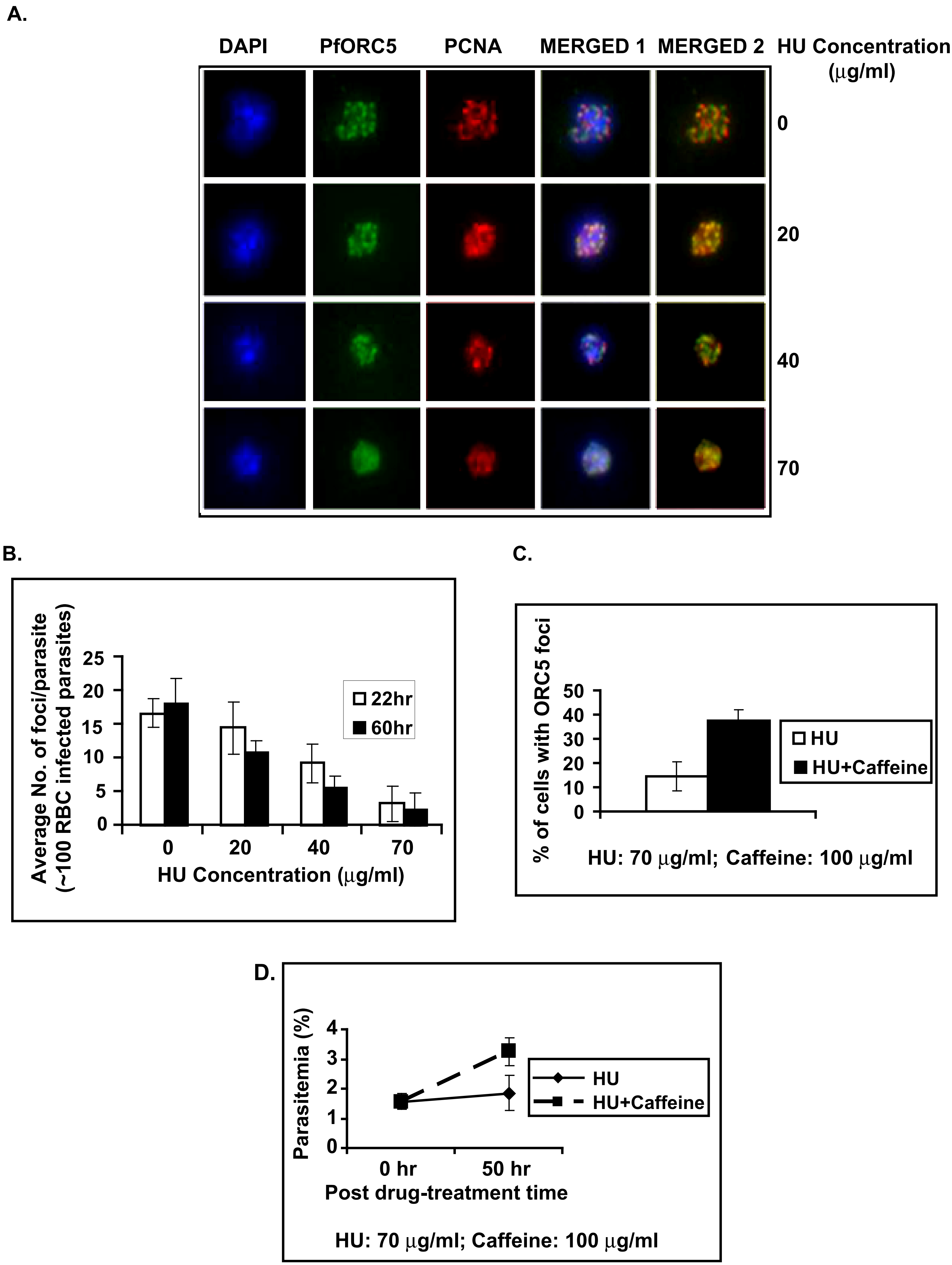

Supplementary Fig. 8
